# Knowledge, Attitudes, and Practices of European Healthcare Professionals towards Hepatitis A and Hepatitis B Vaccination in at-Risk Adults

**DOI:** 10.3390/vaccines11111645

**Published:** 2023-10-26

**Authors:** Dasha Shamarina, Martina Sluga-O’Callaghan, George Kassianos, Alen Marijam, Vaidehi Dave, Eric Davenport, Anar Andani, Desmond Curran, Pavitra Dewda, Robert Steffen

**Affiliations:** 1 GSK, 1300 Wavre, Belgium; 2 RTI Health Solutions, 69009 Lyon, France; 3 Royal College of General Practitioners, London NW1 2FB, UK; 4 British Global & Travel Health Association, London BA1 2SA, UK; 5 RTI Health Solutions, Research Triangle Park, Durham, NC 27709, USA; 6 WHO Collaborating Centre for Travellers’ Health, Epidemiology, Biostatistics and Prevention Institute, University of Zurich, CH-8001 Zurich, Switzerland; 7 Department of Epidemiology, Human Genetics and Environmental Sciences, School of Public Health, University of Texas, Houston, TX 77030, USA

**Keywords:** Europe, guideline, immunization, outbreak, perceived risk, public health, recommendations, survey, vaccine hesitancy

## Abstract

Despite the occurrence of several hepatitis A (hepA) and hepatitis B (hepB) outbreaks in Europe in the last few decades, not all European countries have implemented hepA and hepB vaccinations in their national immunization programs, especially for adults at risk for hepA and/or hepB infection, such as men who have sex with men or patients with chronic liver disease. Currently, little is known on the attitudes of European healthcare professionals (HCPs) towards hepA and hepB vaccinations for at-risk adults. We conducted an online survey among HCPs in Germany, Spain, and the United Kingdom to assess their awareness of and adherence to their national hepA and hepB vaccination guidelines for at-risk adults. Among the 698 HCPs who took the survey, most (91.1%) were familiar with their national vaccination recommendations and always followed them or followed them most of the time when advising or prescribing hepA or hepB vaccines. Major and moderate barriers for recommending or administering such vaccines were the non-disclosure of risk factors by the patient (53.0–57.6%) and the patient’s lack of motivation or knowledge about the risk of the disease (50.3–52.9%). These results may help inform strategies to improve and accelerate hepA and hepB vaccination in European at-risk adults.

## 1. Introduction

Hepatitis A (hepA) and hepatitis B (hepB) are contagious liver infections caused by hepA and hepB viruses (HAV and HBV, respectively) [1,2]. HAV is transmitted mainly via the fecal-oral route, either through direct contact with infected individuals, or by consumption of contaminated water or food [3]. The virus damages the liver through acute infections that, usually, are cleared within several weeks or months [4]; however, in less than 1% of cases, HAV infections may progress to fulminant hepatic failure [5]. HBV is transmitted through contact with infected blood or other body fluids [2], and it can cause hepatic failure through acute and chronic infections that can lead to serious complications such as cirrhosis and hepatocellular carcinoma [6].

Most European countries are considered areas of very low (<2%) endemicity for HAV and HBV infections [7,8]. In 2019, in the European Union (EU) and European Economic Area (EEA) (hereafter referred to as Europe), the mean annual notification rate was 2.2 cases per 100,000 population for confirmed hepA (versus 0.6 in the United Kingdom [UK]) [9] and 0.4 cases per 100,000 population for acute hepB [10]. Nevertheless, in the last two decades, hepA and hepB outbreaks have often been reported across Europe, primarily among men who have sex with men (MSM) [11,12,13,14,15,16]. In addition, in 2021 and 2022, two hepA outbreaks were associated with the consumption of contaminated food in seven European countries [17,18].

Despite the availability of effective and safe monovalent and combined vaccines against HAV and HBV [1,2,19,20], hepA and hepB are still associated with significant morbidity and mortality worldwide [4,6]. The Global Burden of Disease Study estimated that, in 2019, around 159 million people were infected with HAV and 316 million with HBV, and almost 595,000 died of such infections [21,22]. In the same year, the European Centre for Disease Prevention and Control (ECDC) reported 11,370 cases of hepA [9] and 29,996 cases of hepB [10] in Europe. A considerable proportion of these hepA cases (31.6%) occurred in children between 5 and 14 years old while 61.2% of the acute hepB cases and 72.9% of chronic hepB cases were reported in adults between 25 and 54 years old [8].

In May 2016, the World Health Assembly signed the first global health sector strategy on viral hepatitis to eliminate viral hepatitis as a public health threat by 2030 (defined as a 65% reduction in mortality and 90% reduction in new chronic infections compared to the 2015 baseline) [23]. Among other goals, the strategy included vaccination, primarily against hepB (by increasing childhood hepB vaccination coverage from 82% in 2015 to 90% in 2030 and by offering vaccines to people at risk of hepB) and, based on the country context, against hepA [23]. In 2022, the World Health Organization (WHO) and the Centers for Disease Control and Prevention (CDC) in the United States (US) reinforced their recommendations on hepA and hepB vaccination to meet the goals set by the World Health Assembly on viral hepatitis [19,20,23,24,25]. More specifically, the recent guidelines issued by the US CDC extended their recommendation for hepB vaccination to adults aged 19–59 years and adults ≥60 years at risk for hepB and to adults ≥60 years without known risk factors for hepB [25].

The ECDC has not issued any guidance on hepA and hepB vaccination in Europe [26]. While hepB vaccines have been implemented in the childhood immunization programs of most European countries, this is not the case for adult vaccination policies [27]. For example, hepB vaccination is only recommended to at-risk adults in Germany and to previously unvaccinated individuals up to 18 years of age in Spain [28,29]. In the UK, hepB vaccines are recommended to adults at risk for exposure/complications of hepB, including people who inject drugs, individuals with multiple sexual partners, patients with chronic liver disease, travellers to countries with intermediate or high endemicity for hepB who place themselves at risk when abroad, and people with an occupational risk (e.g., healthcare workers) [2].

Most European countries do not recommend hepA vaccination in children [28]. In adults, hepA vaccines are recommended in Greece and the Czech Republic for the general population [28] as well as in Belgium and in the UK for individuals at risk for hepA exposure (e.g., travellers to countries with intermediate or high endemicity for hepA, MSM, and patients with chronic liver disease). In 2017, the WHO Regional Committee for Europe set 2020 as target for 95% hepB vaccination coverage with three doses of hepB vaccine in children, but no goal was defined for adults [24]. Gathering information on the familiarity with and adherence to hepA and hepB vaccination recommendations among healthcare professionals (HCPs) may help improve awareness and compliance with these recommendations. To date, several studies have assessed hepA and hepB vaccination coverage at a national [30,31,32] or European [33,34] level. However, little is known on the attitudes of European HCPs towards hepA and hepB vaccination in at-risk adults despite the latter being the main population affected by hepA and hepB in Europe [17,35]. Therefore, we conducted an online survey among HCPs in three European countries to evaluate their knowledge, practices, and barriers to hepA and hepB vaccination in at-risk adults.

## 2. Materials and Methods

### 2.1. Study Design and Participants

This cross-sectional, web-based survey was carried out between 3 December 2021 and 23 February 2022 among HCPs in Germany, Spain, and the UK. Participants were identified and recruited by Global Perspectives, a survey research company with extensive experience in collecting data from physicians and other HCPs. Participants were recruited by selecting a simple random sample of HCPs by specialty from online HCP panels in the different countries provided by a third-party vendor (RTI Health Solution). The sampling frame included all GPs, FPs, and HCPs available within the country-specific panels constructed and maintained by the third-party vendor who gathered their contact information from multiple sources (e.g., publications, professional organizations, phone calls, etc.). Participants were recruited by sending email invites to a simple random sample of individuals listed in these panels. Those who responded and agreed online to participate in the study and who passed the survey screener (during the timeframe the survey had been online, and the quota had not been reached) were included in the analysis population. Recruitment quotas were set as follows: approximately 225 general practitioners (GPs) or family physicians (FPs) in Germany and Spain and 150 GPs/FPs and 75 HCPs working in sexual health clinics in the UK for a total of 225 HCPs per country and a total study sample size of 675 HCPs.

Eligible participants included HCPs who (i) practiced as licensed GPs or FPs in Germany, Spain, or the UK or as healthcare workers in sexual health clinics (UK only); (ii) were working at least 30 h per week in patient care; (iii) had prescribed, recommended, or administered a hepA or hepB vaccine to an adult aged 18 years or older in the past three months; (iv) were able to complete the survey in the local language (German, Spanish, or English); and (v) electronically consented to participate in the study. Eligible participants were then directed to an informed consent page to review and acknowledge voluntary participation in the study before accessing the self-administered survey.

The survey consisted of a standard questionnaire with closed-ended response choices tailored to address the study objectives and describe the characteristics of the participating HCPs (i.e., years practicing, geography, practice type, patient volume, management of patients with hepA or hepB infections). The survey questionnaire is available in Supplementary Materials. Data collection remained open for 6 weeks or until 225 surveys per country had been completed, whichever occurred first. Participants who completed the survey received financial compensation.

### 2.2. Study Objectives

Primary study objectives included the assessment of HCPs’ knowledge and adoption of the national recommendations for hepA and hepB vaccination of adults, including at-risk groups. Secondary objectives included assessment of HPCs’ practices associated with recommending, prescribing, and/or administering hepA and hepB vaccines to at-risk adults as well as barriers and reasons for not recommending these vaccines. More details on primary and secondary objectives are included in Supplementary Materials.

### 2.3. Study Endpoints

Primary endpoints included the proportion of HCPs who (i) were familiar with national hepA and hepB vaccination recommendations; (ii) followed such guidelines when recommending, prescribing, or administering a hepA or hepB vaccine; (iii) reported following country-specific recommendations always/most of the time when recommending or prescribing hepA or hepB vaccines to adults (aged ≥18 years); (iv) declared extremely important/moderately important to vaccinate at-risk groups against hepA or hepB infections; and (v) recommended a hepA or hepB vaccine to all at-risk groups.

Secondary endpoints included the proportion of HCPs who (i) recommended, prescribed, and/or administered hepA, hepB, and combined hepA/hepB vaccines; (ii) reported reasons for not recommending, prescribing, and/or administering a hepA, hepB, and the combined hepA/hepB vaccine to at-risk groups; (iii) declared using additional resources to inform decision-making when recommending and/or prescribing hepA and hepB vaccines to adults; and (iv) reported potential barriers for not recommending hepA and hepB vaccines to at-risk groups.

### 2.4. Statistical Analysis

No formal hypothesis testing was conducted for this study; therefore, sample size calculations based on a powered hypothesis test were not formally performed to derive the most appropriate sample size. Rather, appropriate sample sizes were determined based on considerations of the sample from the U.S.-based study [36,37], the number of HCPs available within country-specific panels, and the resulting general exact 95% confidence interval (CI) widths for the proposed samples. Although a simple random sample strategy component was present, this sample was one of convenience. Qualified participants were accepted on a ‘first-come’ basis up to the above-mentioned pre-specified quotas.

All participants who gave their consent to participate in the survey and answered at least one survey item were included in the data analyses. These were descriptive and focused on (i) summarizing all questionnaire responses with counts and percentages and (ii) measuring primary and secondary outcomes.

Percentages of HCPs who responded to each questionnaire item were calculated on the total number of HCPs who answered a question excluding those who were asked to skip the question due to a previous response (the sum of these HCPs was listed separately in a row labelled “Not applicable skip pattern” with no percentage). In addition, the sum of HCPs who could answer but did not provide a response was listed in a row labelled “No answer” with a calculated percentage.

Binary outcomes from the derived endpoints of the primary and secondary objectives were evaluated by calculating exact 95% CIs using the Clopper–Pearson method. Secondary endpoints involving barrier scores were assessed by calculating a total barrier score (±standard deviation) by scoring each item from 0 (not at all a barrier) to 3 (major barrier). Barrier scores ranged (i) from 0 to 18 for recommending hepA or hepB vaccines; (ii) from 0 to 15 for administering a hepA vaccine; and (iii) from 0 to 12 for administering a hepB vaccine.

All statistical analyses were performed using Statistical Analysis Software 9.4 (SAS Institute, Inc.; Cary, NC, USA).

## 3. Results

### 3.1. HCP Characteristics

**Figure 1:**
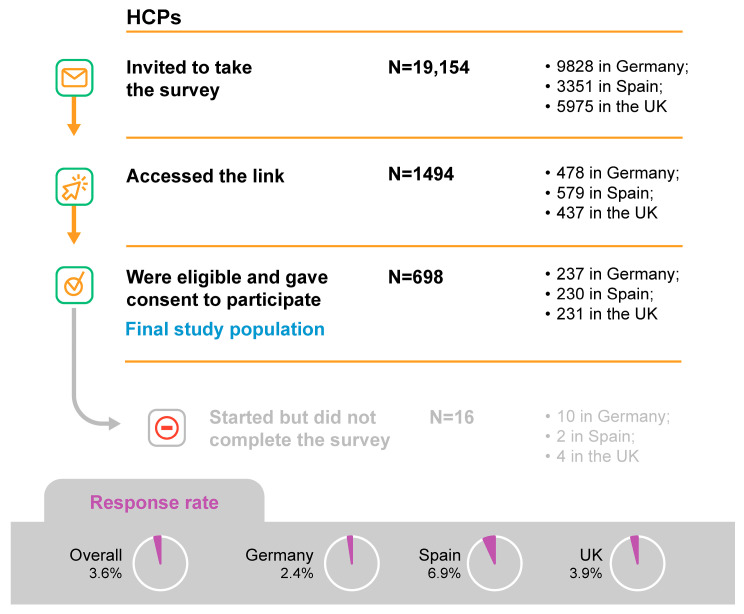
Study population. HCP, healthcare professional; N, number of respondents; UK, United Kingdom.

Of the 19,154 HCPs invited to take the survey, 1494 (7.8%) accessed the link, and 698 (3.6%) were eligible, gave consent to participate, and answered at least one questionnaire item. Of those, 16 HCPs (2.3%) started but did not finish the survey while the remaining 682 completed the survey during the six-week data collection period. The final survey population comprised 698 participants, including 623 GPs/FPs and 75 HCPs working in sexual health clinics (UK only), as reported in Figure 1. Characteristics of the participating HCPs are shown in Table 1.

Across the three countries, the sample of HCPs was relatively diverse in terms of gender, age, and years practicing medicine. In Germany, all GPs/FPs worked in a private practice (solo or group) while in Spain (55.7%) and in the UK (68.6%), they were mostly employed in primary care. The majority of HCPs worked in an urban environment (57.0% in Germany, 86.5% in Spain, and 61.3% in UK HCPs working in sexual health clinic); in the UK, a similar proportion of GPs/FPs worked in an urban (47.4%) and suburban (44.2%) setting. More than 85.0% of participants had received all the nationally recommended vaccines, and 10.0% were partially vaccinated (i.e., they received at least 50% of the nationally recommended vaccines) (Table 1). Details on the geographical region, number of HCPs working in their practice, number of adult patients seen during an average week, and number of adult patients with hepA or hepB seen during all years of practice are provided in the Supplementary Materials (Figures S1–S4).

Overall, most participants reported having encountered over the past month at least one patient at risk for hepA or hepB infection, ranging from 46.1% of HCPs who stated having seen in their practice at least one patient with multiple sexual partners to 59.9% of HCPs who stated having seen at least one healthcare worker possibly exposed to hepB at work (Figure S5).

### 3.2. Awareness of and Adherence to National Guidelines When Recommending, Prescribing, or Administering hepA or hepB Vaccines in Adults

**Figure 2:**
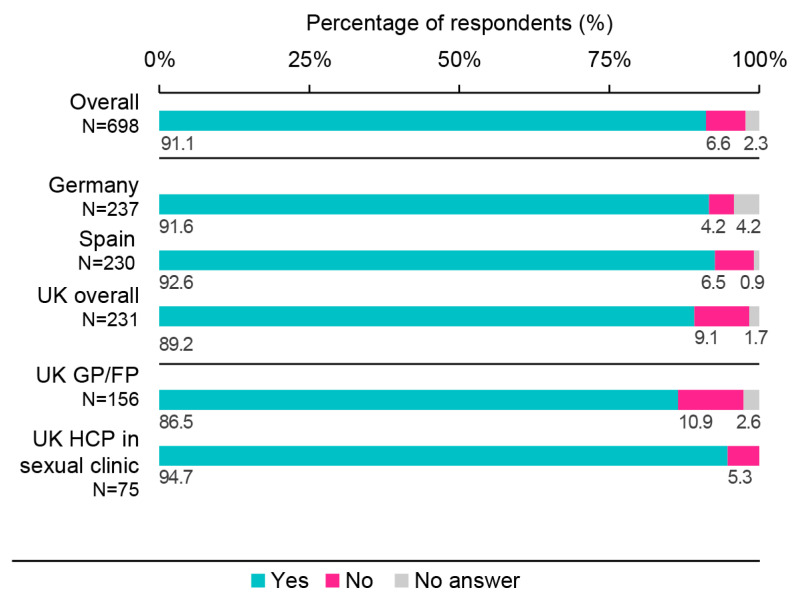
Healthcare professional familiarity with their country vaccination recommendations/guidelines. FP, family physician; GP, general practitioner; HCP, healthcare professional; N, number of respondents; UK, United Kingdom. Survey question: “Are you familiar with the Joint Committee on Vaccination and Immunisation and the NHS (UK only), the Recommendations of the Standing Vaccination Commission (STIKO) (Germany only), the Ministry of Health Recommendations (Spain only)?”.

The majority of HCPs (91.1%; 95% CI: 88.8–93.1%) indicated familiarity with their national vaccination recommendations. More specifically, 91.6% of HCPs in Germany indicated familiarity with the recommendations of the Standing Vaccination Commission, 92.6% of participants in Spain with the Ministry of Health recommendations, and 89.2% of UK participants with the Joint Committee on Vaccination and Immunisation and the National Health Service (NHS) recommendations (86.5% of GPs/FPs and 94.7% of sexual health clinic workers) (Figure 2). More than half of the participants (56.4%) reported receiving training to stay up to date with their country-specific vaccination recommendations and/or guidelines (60.3% in Germany, 62.6% in Spain, 42.9% among UK GPs/FPs, and 53.3% among UK HCPs in sexual health clinics).

**Figure 3:**
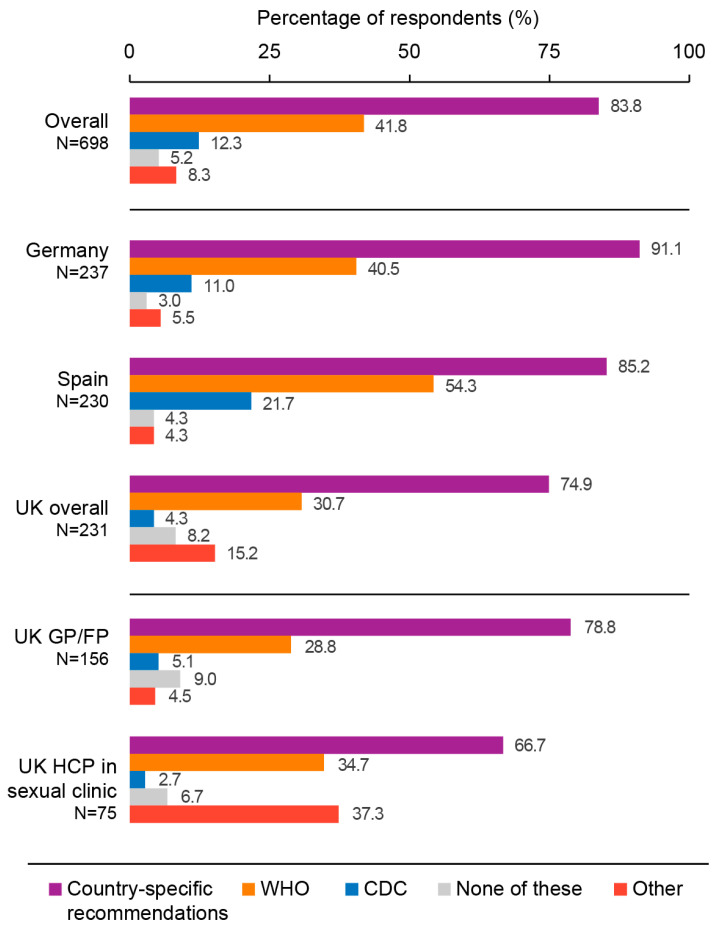
Resources used to decide on recommending, prescribing, or administering a hepatitis A, hepatitis B, or combined hepatitis A/hepatitis B vaccine. CDC, Centers for Disease Control and Prevention; FP, family physician; GP, general practitioner; HCP, healthcare professional; N, number of respondents; UK, United Kingdom; WHO, World Health Organization. Survey question: “When making a decision on recommending, prescribing, or administering a hepatitis A, hepatitis B, and combined hepatitis A/B vaccine to adults (≥18 years of age), what resources do you use?”. Note: Respondents could select more than one answer.

When asked about the resources used to make decisions on recommending, prescribing, or administering a hepA, hepB, or combined hepA/hepB vaccine to adults, the majority of HCPs declared referring to their national vaccination recommendations (74.9% in the UK, 85.2% in Spain, and 91.1% in Germany), followed by the WHO guidelines (30.7% in the UK, 40.5% in Germany, and 54.3% in Spain). The third most consulted resources were the US CDC guidelines in Spain (21.7%) and in Germany (11.0%) and other (unspecified) resources in the UK (15.2%). Overall, only 5.2% of participants indicated to not use any resources when recommending, prescribing, or administering a hepA, hepB, or combined hepA/hepB vaccine to adults (Figure 3).

### 3.3. Knowledge of the Country-Specific Vaccination Guidelines When Recommending, Prescribing, or Administering hepA and hepB Vaccines in at-Risk Adults

**Figure 4:**
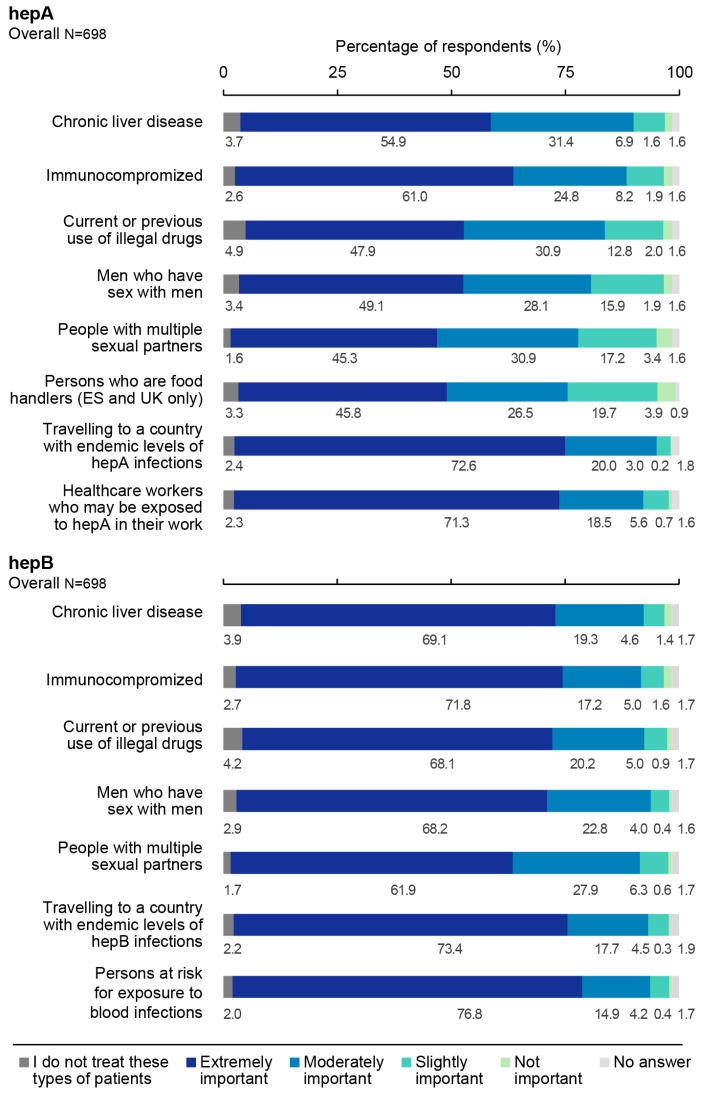
Attitude of healthcare professionals towards hepatitis A and hepatitis B vaccination of specific at-risk groups in the overall sample. ES, Spain; hepA, hepatitis A; hepB, hepatitis B; UK, United Kingdom. HepA survey question: “How important do you think it is that individuals within the following patient populations should get vaccinated for hepatitis A?”. HepB survey question: “How important do you think it is that individuals within the following patient populations should get vaccinated for hepatitis B?”.

Across the three countries, the majority of HCPs considered it extremely important/moderately important to vaccinate against hepA travellers to a country with endemic levels of hepA (92.6%; 95% CI: 90.3–94.5%) and healthcare workers at risk of exposure to hepA (89.8%; 95% CI: 87.3–92.0%). A lower proportion of HCPs considered it extremely important/moderately important to vaccinate people with multiple sexual partners (76.2%; 95% CI: 72.9–79.3%) and food handlers (72.2%; 95% CI: 67.9–76.3%) against hepA (Figure 4). In the UK, a higher proportion of HCPs working in sexual health clinics considered it extremely important to vaccinate most at-risk groups against hepA (except for food handlers and healthcare workers) compared to GPs/FPs. In contrast, a lower proportion of HCPs in sexual health clinic workers (18.7%) considered it extremely important to vaccinate persons at risk of hepA exposure compared to GPs/FPs (76.3%).

Overall, most HCPs considered it extremely important/moderately important to vaccinate against hepB persons at risk for percutaneous, intramuscular, or mucosal exposure to blood (91.7%; 95% CI: 89.4–93.6%), followed by MSM (91.0%; 95% CI: 88.6–93.0%) and travellers to a country with endemic levels of hepB (91.0%; 95% CI: 88.5–93.1%) (Figure 4). In the UK, a higher proportion of HCPs working in sexual health clinics considered it extremely important to vaccinate against hepB in immunocompromised individuals (90.7%) and patients with chronic liver disease (78.7%) than GPs/FPs (65.4% and 66.7%, respectively). Compared to HCPs in Spain (70.0%) and the UK (69.2%), a larger proportion of HCPs in Germany (79.3%) considered it extremely important to vaccinate persons travelling to countries with endemic levels of hepB infection.

**Figure 5:**
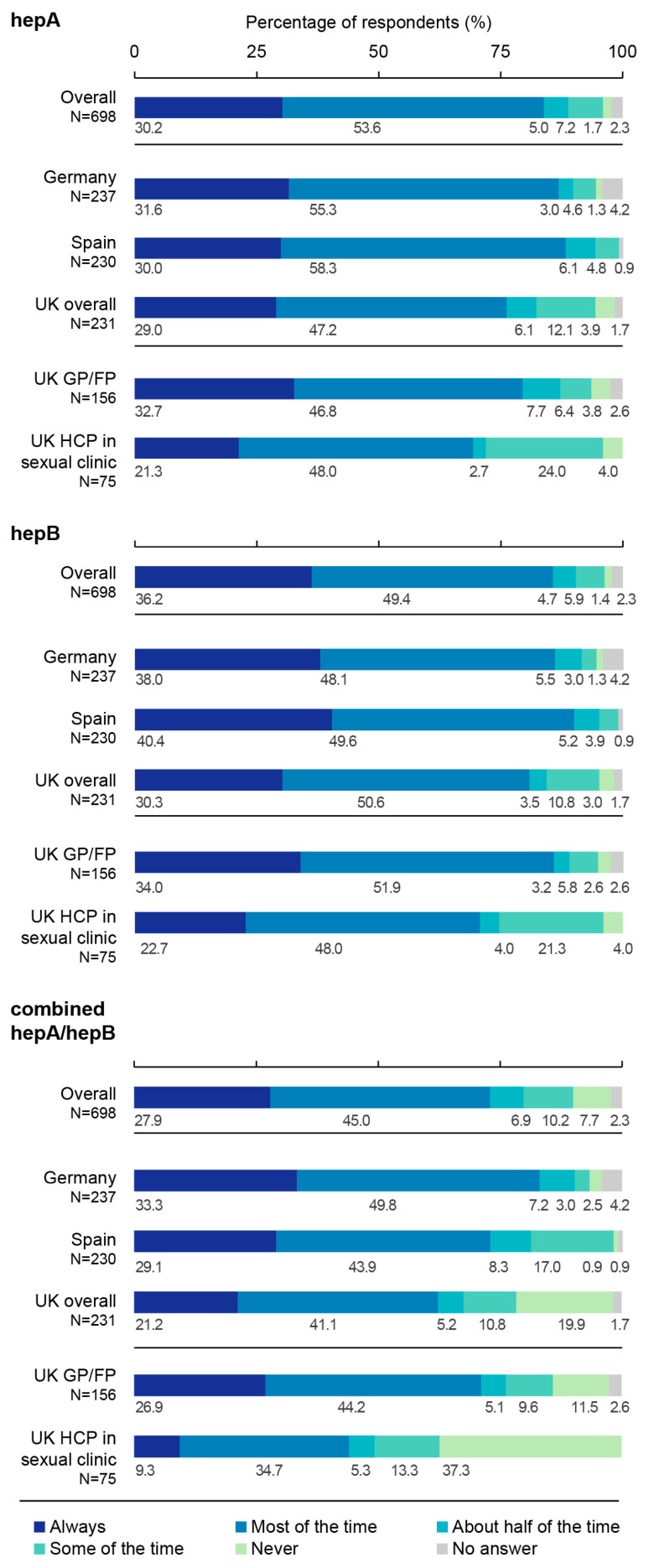
Proportions of healthcare professionals following national vaccination recommendations when recommending or prescribing a hepatitis A, hepatitis B, or combined hepatitis A/hepatitis B vaccine in at-risk adults. FP, family physician; GP, general practitioner; HCP, healthcare professional; hepA, hepatitis A; hepB, hepatitis B; N, number of respondents; UK, United Kingdom. Survey question: “When making a decision on recommending or prescribing a hepatitis A, hepatitis B, or combined hepatitis A/hepatitis B vaccine to adults (≥18 years of age), to what extent do you follow the Joint Committee on Vaccination and Immunisation and the NHS Recommendations (UK only), the Recommendations of the Standing Vaccination Commission (STIKO) (Germany only), the Ministry of Health Recommendations (Spain only)?”.

The majority of participants reported following their national vaccination recommendations always/most of the time when deciding on recommending or prescribing a hepA vaccine in Germany (86.9%), Spain (88.3%), and in the UK (76.2%). Similarly, the majority of HCPs in Germany (86.1%), Spain (90.0%), and in the UK (80.9%) followed their country-specific guidelines always/most of the time when deciding on recommending or prescribing a hepB vaccine. However, compared to the monovalent vaccines, a lower proportion of HCPs reported following their national vaccination recommendations always/most of the time when deciding on recommending or prescribing a combined hepA/hepB vaccine in Germany (83.1%), Spain (73.0%), and in the UK (62.3%). In the UK, the proportion of GPs/FPs who followed their country-specific guidelines always/most of the time when recommending or prescribing a hepA, hepB, or the combined hepA/hepB vaccine were generally higher (79.5%, 85.9%, and 71.1%, respectively) than HCPs working in sexual health clinics (69.3%, 70.7%, and 44.0%, respectively). Finally, a higher proportion of UK HCPs working in sexual health clinics declared that they never follow their national guidelines or only follow them sometimes when recommending or prescribing a hepA (28.0%), hepB (25.3%), and/or combined hepA/hepB vaccine (50.6%) compared to HCPs in Germany (5.9%; 4.3%; 5.5%, respectively), Spain (4.8%; 3.9%; 17.9%, respectively), and UK GPs/FPs (10.2%; 8.4%; 21.1%, respectively) (Figure 5).

### 3.4. Attitudes When Recommending, Prescribing, or Administering a hepA, hepB, or Combined hepA/hepB Vaccine in at-Risk Adults

**Figure 6:**
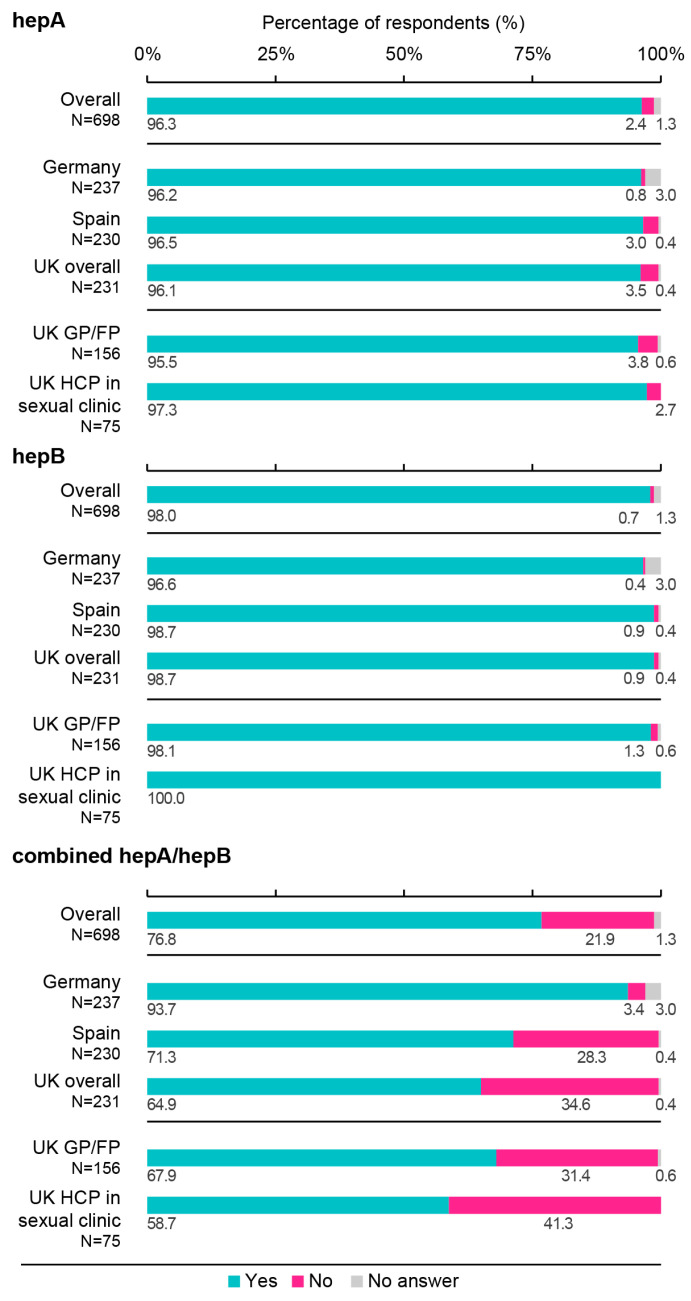
Proportions of healthcare professionals recommending, prescribing, and/or administering hepatitis A, hepatitis B, or combined hepatitis A/hepatitis B vaccines in at-risk adults. FP, family physician; GP, general practitioner; HCP, healthcare professional; hepA, hepatitis A; hepB, hepatitis B; N, number of respondents; UK, United Kingdom. Survey question: “Do you currently recommend, prescribe, and/or administer the following vaccines in your practice: hepatitis A, hepatitis B, or combined hepatitis A/hepatitis B vaccines?”.

In the overall sample, most participants declared to recommend, prescribe, and/or administer hepA (96.3%), hepB (98.0%), and/or combined hepA/hepB (76.8%) vaccines in their practices. These proportions were similar (≥95.5%) for the hepA and hepB vaccine across all countries and specialties, while they differed for the combined hepA/hepB vaccine, ranging from 58.7% (UK HCPs working in sexual health clinics) to 93.7% (GPs/FPs in Germany). The proportion of HCPs who declared recommending a combined hepA/hepB vaccine were higher in Germany (93.7%) than Spain (71.3%) and the UK (64.9%) (Figure 6).

**Figure 7:**
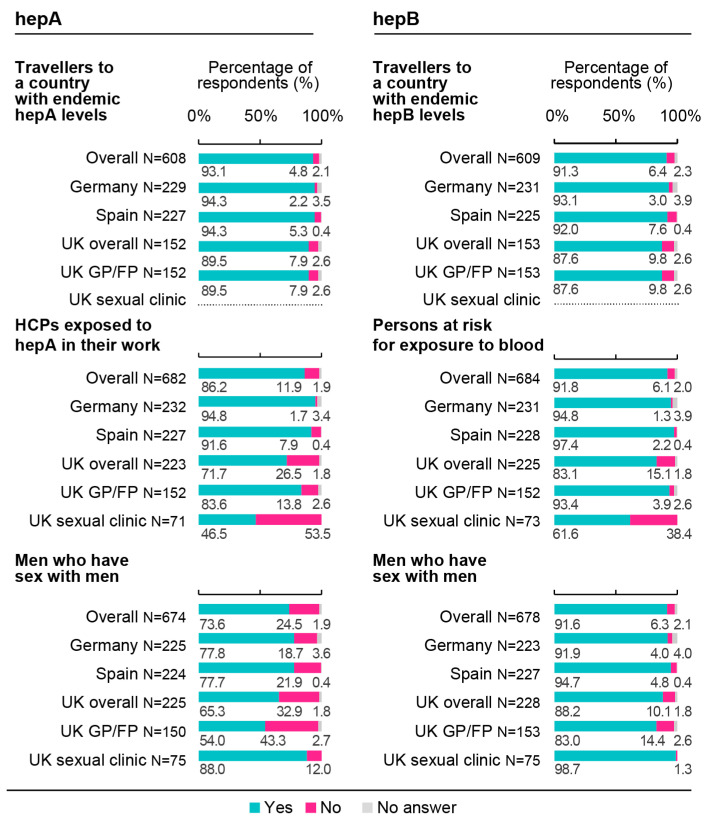
Recommendation practices for hepatitis A and hepatitis B vaccines in at-risk groups. FP, family physician; GP, general practitioner; HCP, healthcare professional; hepA, hepatitis A; hepB, hepatitis B; N, number of respondents; UK, United Kingdom. HepA survey question: “For each patient population (aged ≥18 years) listed, do you recommend a hepatitis A vaccine?”. HepB survey question: “For each patient population (aged ≥18 years) listed, do you recommend a hepatitis B vaccine?”.

Overall, HCPs declared recommending hepA vaccines mostly to travellers to a country with intermediate/high endemic levels of hepA (93.1%; 95% CI: 90.8–95.0%), followed by healthcare workers who may be exposed to hepA in their work duties (86.2%; 95% CI: 83.4–88.7%). Across the three countries, HCPs reported recommending hepB vaccines mostly to persons at risk for percutaneous, intramuscular, or mucosal exposure to blood (e.g., healthcare and public safety staff, such as first responders or emergency personnel) (91.8%; 95% CI: 89.5–93.8%) and MSM (91.6%; 95% CI: 89.2–93.6%) (Figure 7). The proportion of UK HCPs working in sexual health clinics recommending a hepA and/or hepB vaccine were often higher than the other respondent groups, except when recommending such vaccines to food handlers, travellers to a country with intermediate/high endemicity of hepA, and persons at risk for blood exposure (Figure 7 and Figure S6).

### 3.5. Practices and Perceived Barriers When Recommending, Prescribing, or Administering a hepA, hepB, or Combined hepA/hepB Vaccine in at-Risk Adults

**Figure 8:**
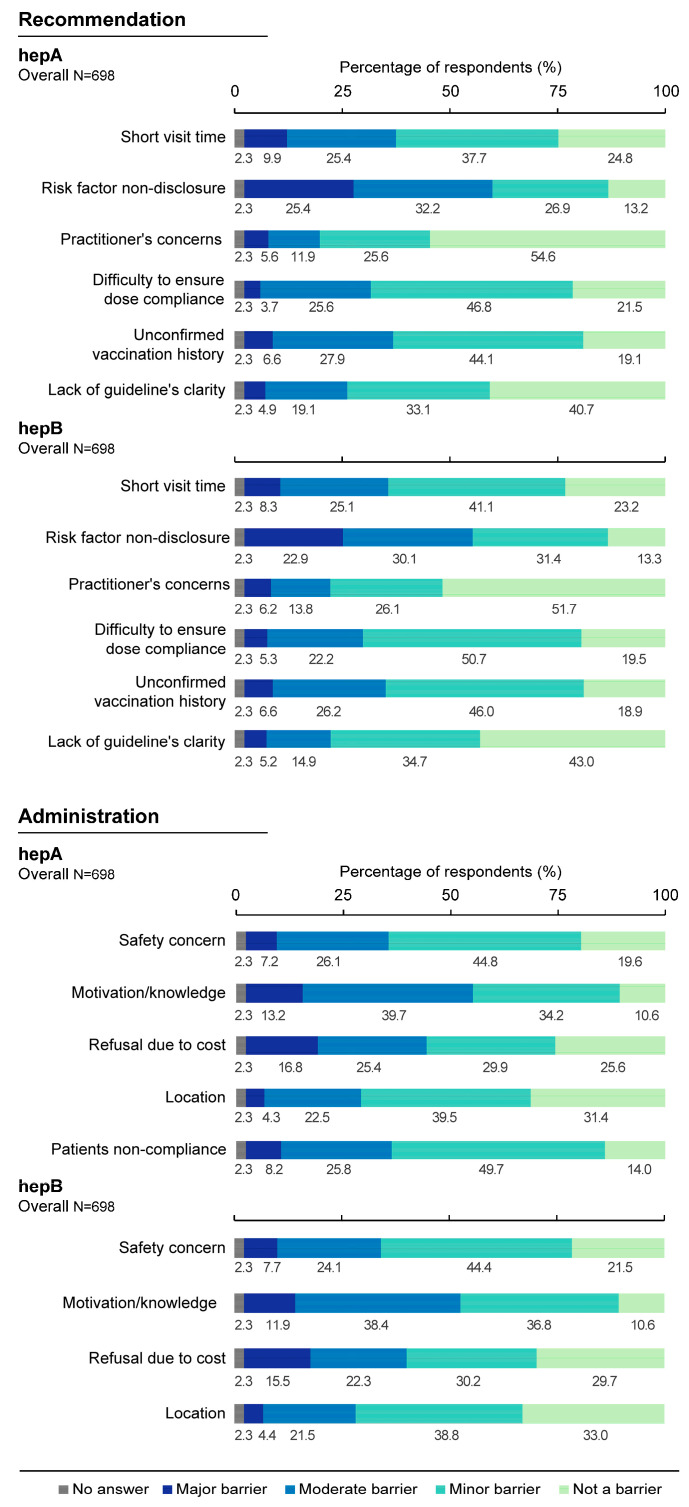
Perceived barriers to recommending or administering a hepatitis A or hepatitis B vaccine in the overall sample. hepA, hepatitis A; hepB, hepatitis B; N, number of respondents. Survey question for recommendation: “To what degree are each of the following a barrier or a concern related to recommending a hepatitis A or a hepatitis B vaccine to your patients at risk of infection?”. Survey question for administration: “To what degree are each of the following a barrier or a concern related to administering a hepatitis A or hepatitis B vaccine to your patients at risk of infection?”.

The non-disclosure of risk factors by the patient to the HCP was the most common major/moderate barrier for recommending hepA (57.6%) and hepB (53.0%) vaccines while a patient’s lack of motivation/willingness or knowledge about the risk of disease was the most common major/moderate barrier for administering hepA (52.9%) and hepB (50.3%) vaccines. The majority of HCPs did not consider their own concerns related to vaccine effectiveness a barrier for recommending hepA (54.6%) or hepB (51.7%) vaccines (Figure 8).

The main reason for not recommending, prescribing, and/or administering a hepA vaccine was the rarity of hepA infections in the HCPs’ practice (50.0% of HCPs in Germany, 100.0% of HCPs in Spain, and 87.5% of HCPs in the UK). In addition, 50.0% of HCPs in Germany declared not to recommend, prescribe, and/or administer such a vaccine for “other (unspecified) reasons”. The main reason for not recommending, prescribing, and/or administering a hepB vaccine was the presence of only a few patients at risk for hepB in Spain (50.0%) and in the UK (50.0%), followed by vaccine refusal by patients in the UK (50.0%) and “other (unspecified) reasons” in Spain (50.0%). In Germany, all HCPs declared that they did not recommend, prescribe, and/or administer such a vaccine for “other (unspecified) reasons”. The main reason for not recommending, prescribing, and/or administering the combined hepA/hepB vaccine was the administration of the individual (non-combined) hepA and hepB vaccines (75.0% in Germany; 67.7% in Spain; 67.5% in the UK).

## 4. Discussion

To our knowledge, this is the first large-scale survey among European HCPs assessing their knowledge, attitudes, practices, and barriers towards hepA and hepB vaccination in at-risk adults. Our results show that the surveyed HCPs were highly familiar with their national vaccination guidelines and always followed them or followed them most of the time when recommending or prescribing hepA and/or hepB vaccines. However, vaccination practices towards at-risk adults differed among HCPs in the three countries, highlighting the need for harmonizing hepA and hepB vaccinations specifically in these populations.

Even though this survey was conducted in countries with different vaccination policies for hepA and hepB in at-risk adults, the majority of HCPs considered all at-risk groups (defined as such in the national, WHO, and/or US CDC guidelines) as important to be vaccinated against hepA and hepB and would recommend both vaccines to most at-risk adults, in line with a recent report from a cross-sectional, web-based survey among HCPs in the US [36]. Most HCPs recommended hepA primarily to travellers to countries with intermediate/high endemic levels of hepA and to healthcare workers who might be in contact with hepA. However, in this survey, MSM were not considered a priority group for hepA vaccination although the hepA outbreaks that occurred in Europe in the last two decades affected this specific risk group [11,12,13,14,15,16]. Thus, there is a need to further raise awareness of the importance of hepA vaccination for this particular population, in line with the recent WHO recommendations [20].

Most participants were familiar with their national vaccination guidelines and always followed them or followed them most of the time when recommending or prescribing hepA, hepB, or combined hepA/hepB vaccines in at-risk adults. In a similar survey conducted in the US, comparable percentages of HCPs recommending and/or prescribing hepA and hepB vaccines were reported [37]. In our survey, most UK HCPs working in sexual health clinics were familiar with their national vaccination recommendations; however, a lower proportion of them declared that they never follow these guidelines or only follow them sometimes when recommending or prescribing a hepA, hepB, and/or combined hepA/hepB vaccine compared to HCPs in Germany, Spain, and UK GPs/FPs. These findings highlight the importance of providing education to HCPs working in sexual health clinics and, more specifically, on the use of the national guidelines.

Only half of HCPs working in sexual health clinics and less than half of GPs/FPs in the UK indicated that they receive trainings to stay up to date on vaccine recommendations. Moreover, HCPs working in sexual health clinics frequently reported using “other (unspecified) resources” for vaccination-informed decision-making. Of note, in the UK, all HCPs must be familiar with the Green Book, which is updated regularly and contains the latest information on vaccines and vaccine procedures [38]. However, the Green Book was not mentioned in our survey, and this could have misled the UK respondents, who may have classified this book among “other resources” and considered it as “self-training” rather than “training”.

Consistent with previous surveys, more than half of HCPs considered the non-disclosure of risk factors by the patient as the main reason for not recommending hepA and hepB vaccines [37,39]. However, since HCPs may not necessarily be aware that their patients might be at risk for hepA and/or hepB (e.g., people travelling to countries with intermediate/high endemicity for hepA and/or hepB), they could proactively inform them on the possibility of hepA and/or hepB vaccination by using, for example, information leaflets displayed in their waiting rooms.

Compared to the UK, a higher proportion of HCPs in Germany and Spain reported out-of-pocket cost as the second major or moderate barrier for administering hepA and hepB vaccines in at-risk adults. In the UK, several at-risk groups (listed in the Green Book) can get hepA and hepB vaccine free of charge on the NHS [40,41]. In Spain and in Germany, on the other hand, the list of at-risk individuals for hepA and hepB benefitting from vaccination at no cost is more limited [29,42]. However, different policies are applied by the NHS when reimbursing the costs of hepA, hepB, and combined hepA/hepB vaccines [40,41], and this may be the reason why a lower proportion of HCPs in the UK declared that they recommend, prescribe, and/or administer the combined hepA/hepB vaccines than in Germany and Spain. In the US, where hepA/hepB vaccination is generally not reimbursed, out-of-pocket cost was also identified as an important barrier for vaccination [37]. Reducing financial barriers to the administration of such vaccines in at-risk groups should therefore be among the strategies adopted by each country to increase hepA and hepB vaccination coverage and prevent new infections, especially in view of the goal set by the WHO for reducing viral hepatitis by 2030. 

The strengths of this study are the large sample size and the inclusion of HCPs from three representative countries, not only in terms of population, but also of vaccination practices against hepA and hepB. Moreover, this survey targeted HCPs who had recommended, prescribed, and/or administered hepA and hepB vaccines in the three months prior to completing the survey, that is, GPs, FPs, and healthcare workers in sexual health clinics who were really involved in vaccination practices against hepA and/or hepB.

This study has several limitations, including a potential selection bias since the participants were recruited through a panel of members whose knowledge of and adherence to country-specific vaccination recommendations may be different from that of HCPs not included in the panel. Furthermore, the underrepresentation of certain categories of HCPs in Spain and in the UK (those practicing in a solo/group private practice, hospital, or outpatient clinic) as well as the very low HCP response rates typical of web-surveys [43] may have increased the likelihood of selection bias. Finally, analyses were reported from aggregated data among GPs/FPs and HCPs working in sexual health clinics (UK only), which may have biased conclusions specific to one specialty.

## 5. Conclusions

**Figure 9:**
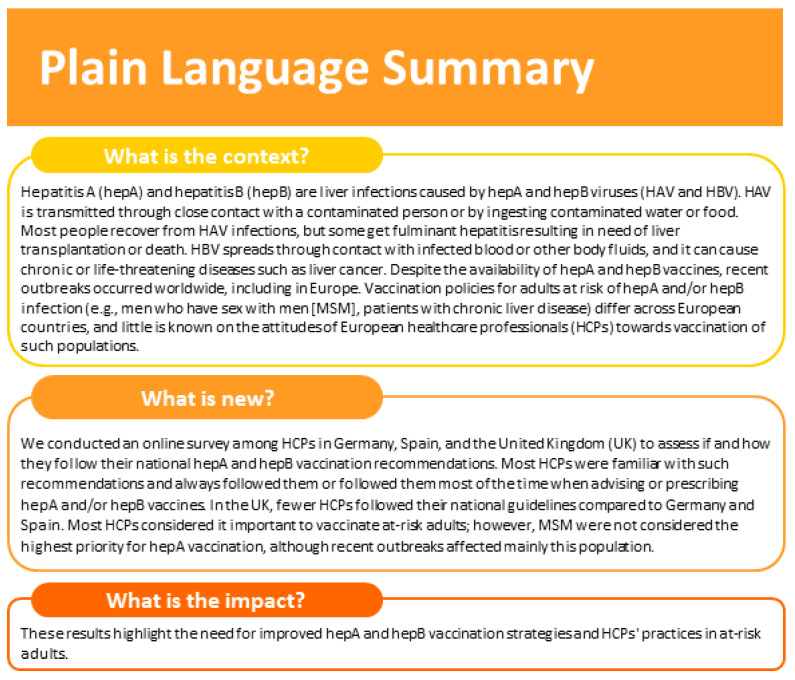
Plain Language Summary.

The results of this survey show that the majority of HCPs in Germany, Spain, and the UK are familiar with and adhere to their national guidelines when recommending, prescribing, and/or administering hepA and hepB vaccines in at-risk adults. Most of them consider vaccinating such patients against hepA and hepB important, but hepA and hepB vaccination policies highly differ among these countries. Not all HCPs recommend such vaccines to all at-risk groups. There is a need to provide HCPs with more detailed education on vaccination for certain at-risk groups, particularly to enable them to identify those hiding their risk factors voluntarily or just because they are not asked the right question. It is also essential that HCPs take the time for hepA and hepB vaccination for each and every patient. The implementation of the recent recommendations issued by the WHO and the US CDC on the efficacy of the single hepA vaccination and the universal administration of the hepB vaccine as well as proactive participation of clinicians and public health services in recommending hepA/hepB vaccination to the appropriate patients could help increasing vaccination coverage and achieving the ambitious goal to eliminate viral hepatitis as a global health threat by 2030. A plain language summary is provided in Figure 9.

## Figures and Tables

**Table 1:** Characteristics of healthcare professionals participating in the survey.

Category, n (%)	Germany (N = 237)	Spain (N = 230)	UK (N = 231)	
			GP/FP (N = 156)	HCP in Sexual Health Clinic (N = 75)
Gender				
Female	76 (32.1)	75 (32.6)	45 (28.8)	43 (57.3)
Male	149 (62.9)	146 (63.5)	99 (63.5)	31 (41.3)
Genderfluid	1 (0.4)	2 (0.9)	1 (0.6)	0 (0.0)
Non-binary	0 (0.0)	0 (0.0)	1 (0.6)	0 (0.0)
Prefer not to answer	1 (0.4)	5 (2.2)	6 (3.8)	1 (1.3)
No answer	10 (4.2)	2 (0.9)	4 (2.6)	0 (0.0)
Age (years)				
18–29	0 (0.0)	22 (9.6)	0 (0.0)	4 (5.3)
30–39	18 (7.6)	53 (23.0)	29 (18.6)	27 (36.0)
40–49	61 (25.7)	58 (25.2)	63 (40.4)	34 (45.3)
50–59	96 (40.5)	74 (32.2)	42 (26.9)	9 (12.0)
60–69	48 (20.3)	17 (7.4)	11 (7.1)	1 (1.3)
≥70	0 (0.0)	0 (0.0)	2 (1.3)	0 (0.0)
Prefer not to answer	4 (1.7)	4 (1.7)	5 (3.2)	0 (0.0)
No answer	10 (4.2)	2 (0.9)	4 (2.6)	0 (0.0)
Years practicing medicine				
≤5	1 (0.4)	15 (6.5)	7 (4.5)	7 (9.3)
6–10	17 (7.2)	42 (18.3)	20 (12.8)	20 (26.7)
11–15	53 (22.4)	39 (17.0)	38 (24.4)	20 (26.7)
16–20	63 (26.6)	49 (21.3)	37 (23.7)	23 (30.7)
21–25	46 (19.4)	45 (19.6)	32 (20.5)	3 (4.0)
>25	47 (19.8)	38 (16.5)	18 (11.5)	2 (2.7)
No answer	10 (4.2)	2 (0.9)	4 (2.6)	0 (0.0)
Primary work environment				
Private practice, solo	117 (49.4)	12 (5.2)	12 (7.7)	0 (0.0)
Private practice, group	120 (50.6)	20 (8.7)	26 (16.7)	0 (0.0)
Public primary care	0 (0.0)	128 (55.7)	107 (68.6)	0 (0.0)
Hospital, inpatient service	0 (0.0)	45 (19.6)	3 (1.9)	3 (4.0)
Hospital, outpatient service	0 (0.0)	25 (10.9)	7 (4.5)	26 (34.7)
Sexual health clinic ^a^	NA	NA	1 (0.6)	46 (61.3)
Practice setting ^b^				
Rural	32 (13.5)	12 (5.2)	12 (7.7)	1 (1.3)
Suburban	67 (28.3)	18 (7.8)	69 (44.2)	28 (37.3)
Urban	135 (57.0)	199 (86.5)	74 (47.4)	46 (61.3)
No answer	2 (0.8)	1 (0.4)	0 (0.0)	0 (0.0)
Vaccination status as per national recommendation				
Fully vaccinated ^c^	187 (78.9)	212 (92.2)	136 (87.2)	64 (85.3)
Partially vaccinated ^d^	33 (13.9)	14 (6.1)	12 (7.7)	11 (14.7)
Not fully vaccinated ^e^	7 (3.0)	2 (0.9)	4 (2.6)	0 (0.0)
No answer	10 (4.2)	2 (0.9)	4 (2.6)	0 (0.0)

FP, family physician; GP, general physician; HCP, healthcare professional; N, total number of respondents; n (%), number (percentage) of respondents in a particular category; NA, not available; UK, United Kingdom. ^a^ Question asked in the UK only; ^b^ Primary work environment; ^c^ Received all nationally recommended vaccines, including but not limited to influenza, diphtheria tetanus pertussis, and COVID-19 vaccines; ^d^ Received ≥ 50% of the nationally recommended vaccines; ^e^ Received < 50% of the nationally recommended vaccines.

## Data Availability

The data presented in this study are available upon request from the corresponding author.

## Supplementary Materials

The following supporting information can be downloaded at: https://www.mdpi.com/article/10.3390/vaccines11111645/s1, Study objectives; Figure S1: Geographical regions of healthcare professionals; Figure S2: Proportions of healthcare professionals (HCPs) based on the number of HCPs working in the practice, including respondents; Figure S3: Proportions of healthcare professionals based on the number of adult patients seen in practice during an average week; Figure S4: Proportions of healthcare professionals based on the number of adult patients with hepatitis A or hepatitis B infections seen in all years of practice; Figure S5: Proportions of healthcare professionals based on the number of at-risk adults seen over the past month in the overall sample; Figure S6: Recommendation practices for hepatitis A and hepatitis B vaccines in other at-risk groups; Survey Questionnaire.
