# Supplementary material for: Knowledge, Attitudes, and Practices of European Healthcare Professionals towards Hepatitis A and Hepatitis B Vaccination in at-Risk Adults
Source: Vaccines (Basel). 2023 Oct 26;11(11):1645. doi: 10.3390/vaccines11111645 (PMC10675089; doi:10.3390/vaccines11111645)
Supplement: Supplementary file 1 [file vaccines-11-01645-s001.zip › vaccines-2628401-supplementary.pdf]

Supplementary Materials

Study objectives

The primary objective of this study were:

- To evaluate healthcare professionals (HCPs)' use and general knowledge of the country-specific vaccination recommendations for decision-making in recommending, prescribing, or administering hepatitis A (hepA) or hepatitis B (hepB) vaccines to adult patients
- To evaluate HCPs' knowledge regarding recommending, prescribing, or administering a hepA vaccine to patients at risk of hepA infection or complications of hepA disease, with a focus on patients with chronic liver disease, immunocompromised individuals, illegal drug users, men who have sex with men (MSM), people with multiple sexual partners, travellers to a country with intermediate/high endemic levels of hepA infection (e.g., Italy, Turkey, Spain, Russia, South America, Africa, parts of Asia), and healthcare workers who may be exposed to hepA in their work duties (e.g., exposure to stools)
- To evaluate HCPs' knowledge regarding recommending, prescribing, or administering a hepB vaccine to patients at risk of hepB infection or complications of hepB disease, with a focus on patients with chronic liver disease, immunocompromised individuals, illegal drug users, MSM, people with multiple sexual partners, travellers to a country with intermediate/high endemic levels of hepB infection (e.g., Romania, Latvia, Albania, Southeast Asia, Northern Canada), and people at risk for percutaneous, intramuscular, or mucosal exposure to blood (e.g., healthcare and public safety staff, such as first responders or emergency personnel)
- To evaluate HCPs' self-reported adherence to the country-specific recommendations regarding recommending, prescribing, or administering hepA and hepB vaccines to patients at risk of hepA and hepB infections or at risk of complications of hepA or hepB disease, with a focus on the at-risk patient populations for each vaccine

The secondary objectives were:

- To identify HCPs' practices associated with recommending, prescribing, and/or administering hepA vaccines to all patients at risk of hepA infections or complications of hepA disease
- To identify HCPs' practices associated with recommending, prescribing, and/or administering hepB vaccines to all patients at risk of hepB infections or complications of hepB disease
- To identify perceptions and reasons why HCPs may not recommend hepA and hepB vaccines in at-risk adults
- To evaluate barriers related to recommending, prescribing, or administering a hepA or hepB vaccine in at-risk adults

Supplemental figures

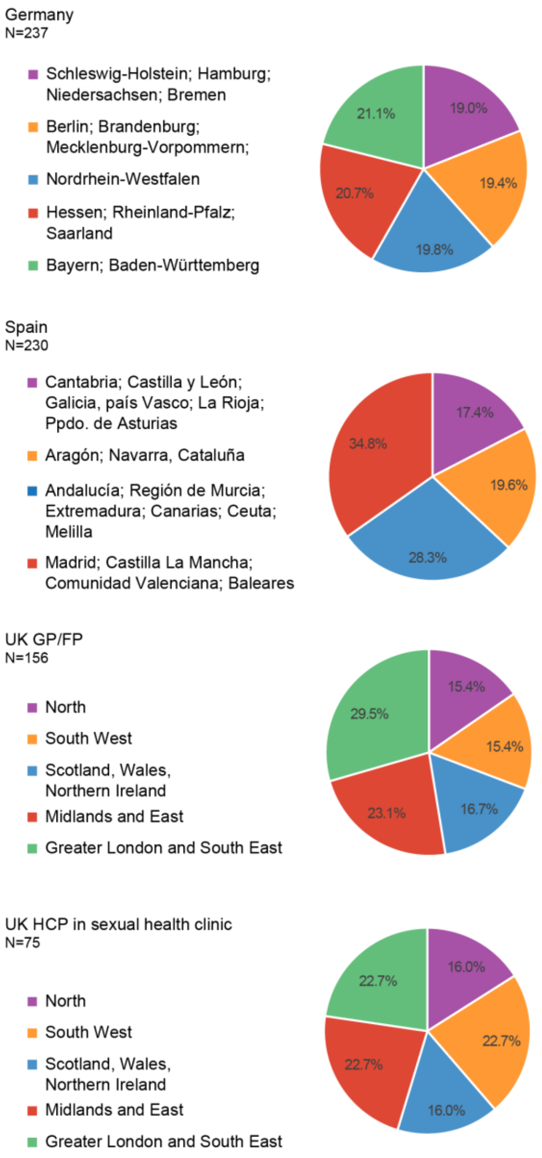

Supplemental Figure S1. Geographical regions of healthcare professionals. FP, family physician; GP, general physician; HCP, healthcare professional; N, total number of respondents; UK, United Kingdom.

Survey question: "In which region is your practice located? If your practice is located in more than one region, please select the region where you consider your primary practice to be located".

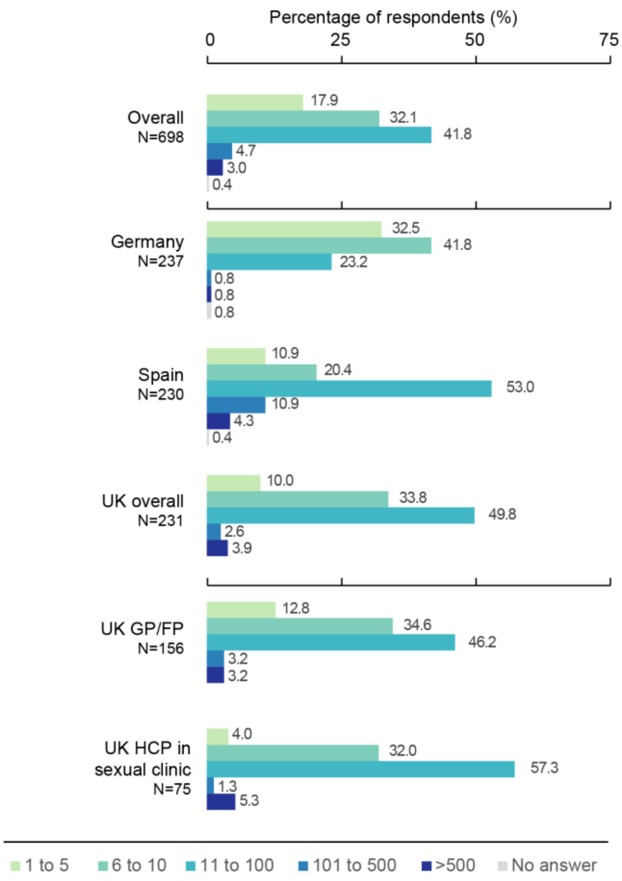

Supplemental Figure S2. Proportions of healthcare professionals (HCPs) based on the number of HCPs working in the practice, including respondents. FP, family physician; GP, general physician; HCP, healthcare professional; N, total number of respondents; UK, United Kingdom.

Survey question: “How many healthcare professionals (e.g., physicians, physician assistants, and/or nurse practitioners) are in your practice (including yourself)?”.

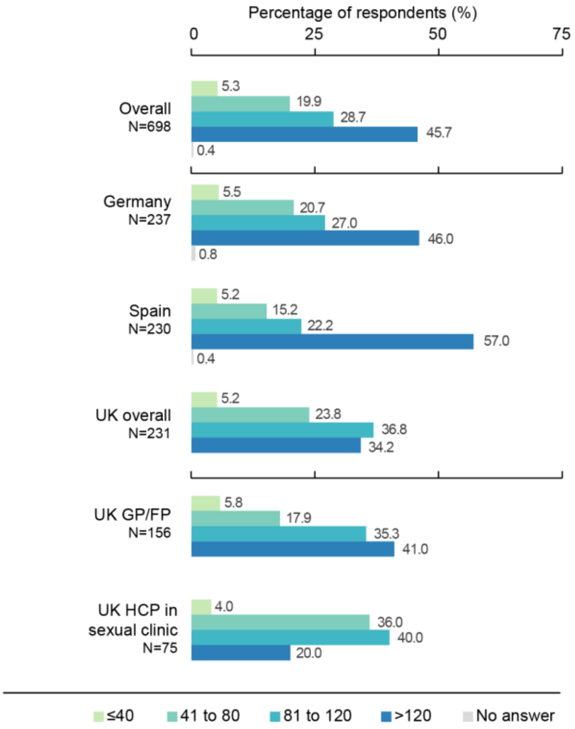

Supplemental Figure S3. Proportions of healthcare professionals based on the number of adult patients seen in practice during an average week. FP, family physician; GP, general physician; HCP, healthcare professional; N, total number of respondents; UK, United Kingdom.

Survey question: "During an average week (5 working days), how many adult patients (≥18 years of age) do you evaluate and/or treat face to face in your practice for any medical condition?".

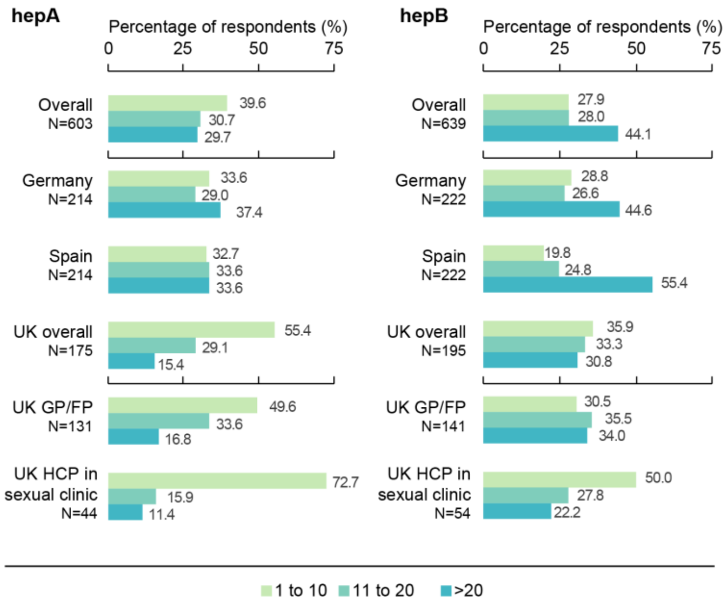

Supplemental Figure S4. Proportions of healthcare professionals based on the number of adult patients with hepatitis A or hepatitis B infections seen in all years of practice. FP, family physician; GP, general physician; HCP, healthcare professional; hepA, hepatitis A; hepB, hepatitis B; N, total number of respondents; UK, United Kingdom.

HepA survey question: "In all of your years of practice, approximately how many adult cases of hepatitis A infection have you encountered?". This question was asked to HCPs who answered 'Yes' to the following question: "Have you ever encountered any adult patients (≥18 years of age) who have had or currently have a hepatitis A infection?". HepB survey question: "In all of your years of practice, approximately how many adult cases of hepatitis B infection have you encountered?". This question was asked to HCPs who answered 'Yes' to the following question: "Have you ever encountered any adult patients (≥18 years of age) who have had or currently have a hepatitis B infection?".

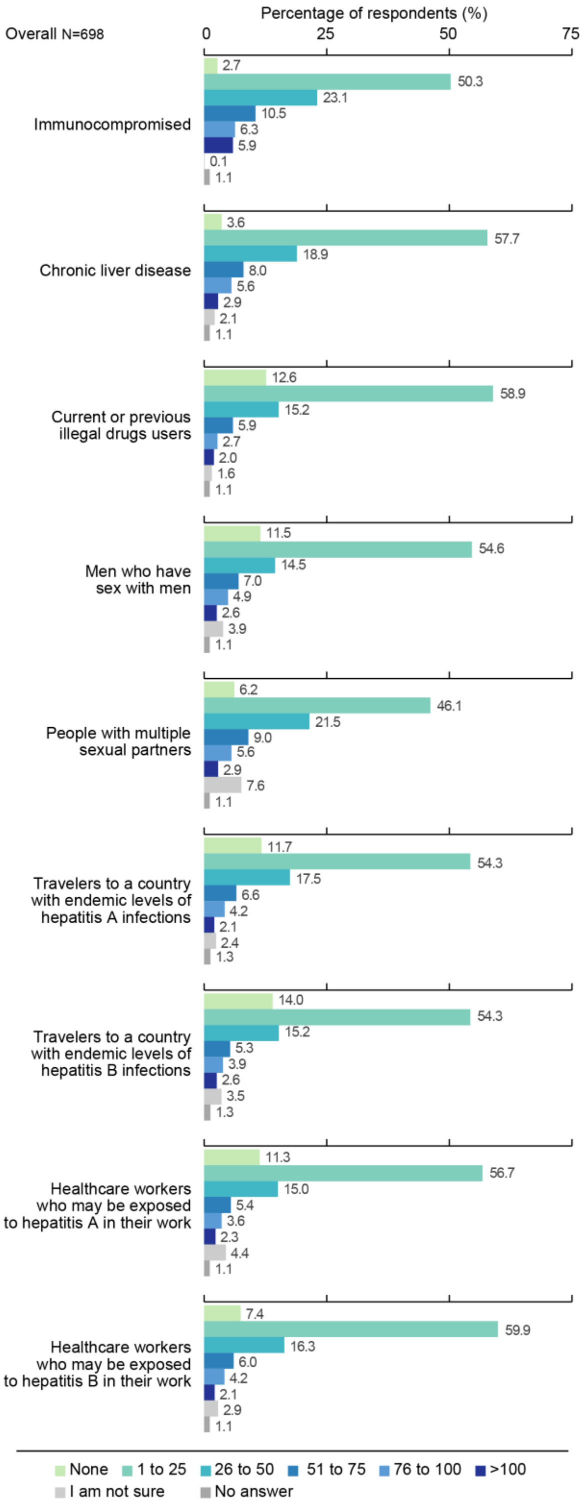

Supplemental Figure S5. Proportions of healthcare professionals based on the number of at-risk adults seen over the past month in the overall sample. N, total number of respondents.

Survey question: "Over the past month, please provide an estimate of the average number of patients that you have seen in your practice".

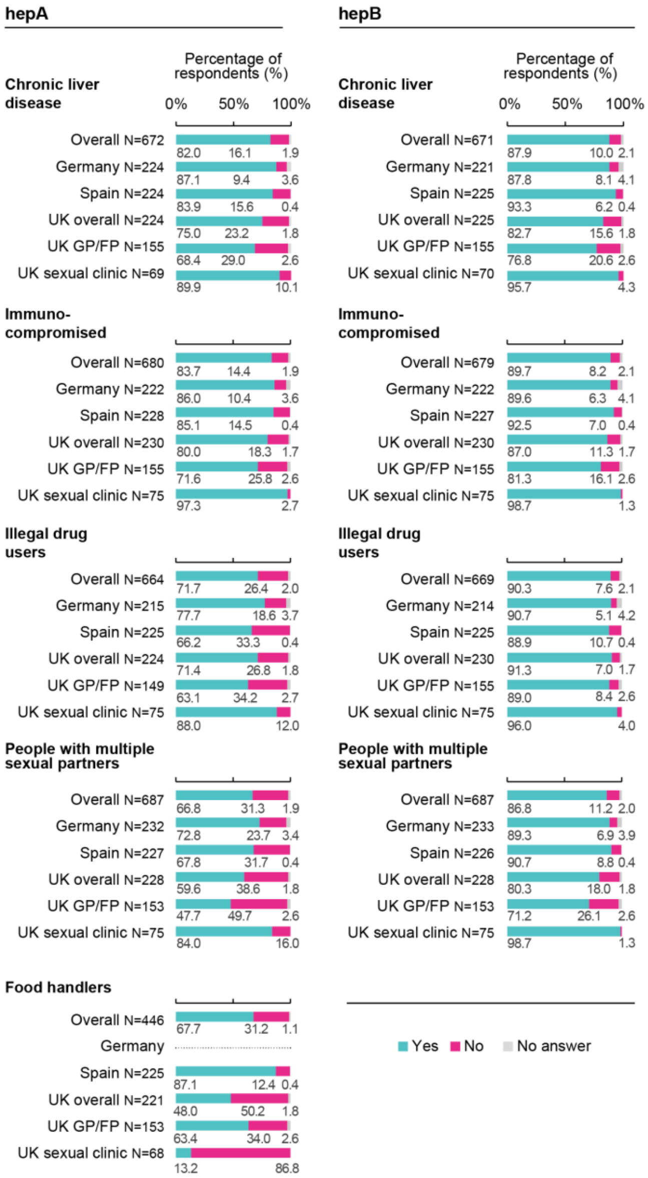

Supplemental Figure S6. Recommendation practices for hepatitis A and hepatitis B vaccines in other at-risk groups. FP, family physician; GP, general practitioner; HCP, healthcare professional; hepA, hepatitis A; hepB, hepatitis B; N, number of respondents; UK, United Kingdom.

HepA survey question: “For each patient population (aged ≥18 years) listed, do you recommend a hepatitis A vaccine?”. HepB survey question: “For each patient population (aged ≥18 years) listed, do you recommend a hepatitis B vaccine?”.

Knowledge, Attitudes, and Practises (KAP) of Healthcare Providers: Questionnaire Regarding Use of Hepatitis A and B Vaccines Study

[Do not display the title to HCPs completing the questionnaire for the screener]

We welcome you to this research questionnaire and thank you for your interest in this survey.

The purpose of this study is to learn about healthcare providers' knowledge of vaccines and recommendations for vaccination of specific at-risk groups and to assess barriers related to vaccination practices.

Screening Questions

If you are interested in participating in the study, please answer the following questions to determine if you qualify.

S1. Are you a licensed healthcare provider and currently practising medicine in [Display country specific option: United Kingdom, Germany, or Spain]?

[Single answer]

☐ Yes

☐ No [Ineligible; terminate immediately]

S2. In which region is your practice located? If your practice is located in more than one region, please select the region where you consider your primary practice to be located.

[Single answer]

	**UK**	**Germany**	**Spain**
1	North	Schleswig-Holstein; Hamburg; Niedersachsen; Bremen	Madrid; Castilla La Mancha; Comunidad Valenciana; Baleares
2	Midlands and East	Nordrhein-Westfalen	Aragón; Navarra, Cataluña
3	Greater London and South East	Hessen; Rheinland-Pfalz; Saarland	Cantabria; Castilla y León; Galicia, país Vasco; La Rioja; Ppdo. de Asturias
4	South West	Bayern; Baden-Württemberg	Andalucía; Región de Murcia; Extremadura; Canarias; Ceuta; Melilla
5	Scotland, Wales, Northern Ireland	Berlin; Brandenburg; Mecklenburg-Vorpommern; Thüringen; Sachsen; Sachsen-Anhalt	

S3. Please indicate your primary medical profession.

[Single answer]

- ☐ General physician (GP) or family physician (FP)
- ☐ Healthcare provider (HCP) working in a sexual health clinic [UK only]
- ☐ Other healthcare provider [Ineligible; terminate immediately]

S4. Where is your primary work environment?

[Single answer]

- ☐ Private practice, solo
- ☐ Private practice, group
- ☐ Public primary care [ES and UK only]
- ☐ Sexual health clinic [UK only]
- ☐ Other

[All responses are eligible]

S5. On average, how much time do you work per week in patient care?

[Single answer]

- ☐ Less than 30 hours [Ineligible; display the ineligible screen]
- ☐ 30 hours or more (full-time employee)

S6. Over the past 3 months, have you recommended, prescribed, and/or administered any of the following vaccines to at least 1 adult patient? Please select all that apply. [Please randomise the responses except the last one; always anchor at bottom]

[Multiple answers]

- ☐ Hepatitis A [Hepatitis A must be selected to qualify]
- ☐ Hepatitis B [hepatitis B must be selected to qualify]
- ☐ Combined hepatitis A and hepatitis B
- ☐ Coronavirus

☐ Measles, mumps, rubella/measles, mumps, rubella, varicella (MMR/MMRV)

☐ Tetanus and diphtheria (Td)

☐ Tetanus, diphtheria, and pertussis (Tdap-)(IPV)

☐ Herpes Zoster

☐ Influenza

☐ Other vaccines (e.g., rabies, travel vaccines)

I have not prescribed, recommended, and/or administered any of these vaccines. [Exclusive. Do not randomise, always show at bottom]

Ineligible Screen

Thank you very much for answering these questions. Unfortunately, based on your responses, you are not eligible to complete this survey. Thank you again for your time and interest. [Termination point]

Eligible Screen

Thank you very much for answering these questions. Based on your responses, you are eligible to complete this survey questionnaire. [Continue to informed consent]

[Display the title to HCPs]

Informed Consent

Please read this information carefully before deciding to participate. If you choose to take part in this research study, you will need to provide consent.

This research study is being conducted by RTI Health Solutions (RTI-HS) on behalf of GlaxoSmithKline Biologics, SA (GSK). RTI-HS is a business unit of RTI International, a not-for-profit research organisation with headquarters in Research Triangle Park, North Carolina in the United States. RTI-HS has contracted with Global Perspectives to recruit individuals from their healthcare provider panel, screen these individuals for eligibility, and collect data from consented participants.

What is the purpose of the study?

The purpose of this online survey is to assess your knowledge of hepatitis A and B vaccine recommendations for vaccination of specific at-risk groups. In addition, this survey will evaluate barriers related to your vaccination practises.

How many other people will be in the study, and how long will participation in the study last?

There will be about 675 healthcare professionals enrolled in this study, about 225 each in the United Kingdom, Germany, and Spain. The online survey will be administered only once and will take approximately 20 minutes to complete.

What are possible outcomes of being in this study?

Your responses and the information we collect are very important because they will help researchers to understand healthcare providers' awareness, practises, and barriers related to vaccinating adults for prevention of hepatitis A and B infections. There may be possible benefit to broader public health in terms of greater awareness and accessibility to prevention strategies.

Is being in the study voluntary?

Yes, your decision to take part in this research study is completely voluntary. You can stop at any point after you begin the survey. No new information will be gathered after that point. However, we may continue to use the data that you provided before withdrawing consent, as described in this informed consent.

Will I be paid for taking part in this study?

We value your time and opinions and will provide compensation as noted in your email invitation to participate in this survey.

What are the risks and possible discomforts of being in this study?

There is a potential risk of disclosure of your responses. Every effort will be made to protect your information, but this cannot be guaranteed. This is a minimal risk research study; your contact information will not be provided to RTI-HS from Global Perspectives. We will take every precaution to protect your privacy. Your responses to the questions in this survey will be reported only in aggregate and anonymous form. At the completion of the study, the anonymised analysis data set will be available to GSK and will not include any information on individual participants. RTI-HS, Global Perspectives, and GSK will not be able to link your answers to you, and your answers will not be linked to your name in any report or publication. The data you provide will be hosted on a secure server and will be encrypted.

Anonymised responses (not including your name and contact information) will be shared with RTI-HS researchers in the US for data analysis and storage purposes. Your answers will not be linked to your name in any report or publication. The risk of participation in this study relates to data security and is expected to be minimal given the strict confidentiality and security procedures in place.

If you have questions regarding your data or wish to access your data or to withdraw your consent as permitted under the data protection laws of your country, please contact Global Perspectives at [insert Global Perspectives emails for each country]**.**

Please note that once your data has been aggregated with responses from other participants, it will not be possible to identify your data separately or remove it from the

study. The results of this study may be used by GSK for scientific presentations and/or publication in a peer-reviewed journal, as well as to inform internal decision making for future research.

Whom should I contact about my rights or if I have questions?

If you have any questions about the collection and use of information about you, you may call the Global Perspectives representative, [insert name of Global Perspectives contact person in each country], at [insert email of Global Perspectives contact person in each country].

If you have any questions about the study, you may email the RTI-HS project leader, Martina Sluga-O'Callaghan, at mocallaghan@rti.org. Every attempt will be made to answer any messages within 3 business days of receipt.

Consent

Please indicate below if you agree to participate in the current study.

☐ Yes, I have read the study information provided as part of the questionnaire and agree to participate in this study

☐ No, I do not agree to participate in this study [Terminate]

[If consent = "Yes, I have read the study information provided as part of the questionnaire and agree to participate in this study," continue.]

[If consent = "No, I do not agree to participate in this study," then display "You have clicked 'No, I do not agree to continue participate in this study.' Are you sure you don't want to participate?" then re-present options. On second presentation, if 'no' is selected, then display "You have indicated that you do not agree to participate in the study. Thank you for your time." Terminate survey.]

Healthcare Provider Questionnaire

Thank you very much for answering these questions. Based on your responses, you are eligible to complete this survey.

The first few questions ask about your experience as a healthcare provider and the practice environment in which you work, followed by more detailed questions about your current immunisation practices for prevention of hepatitis A and B infections.

1. Which of the following categories best describes the location of your primary work environment?

[Single answer]

☐ Rural

☐ Suburban

☐ Urban

☐ Other

2. How many healthcare providers (e.g., physicians, physician assistants, and/or nurse practitioners) are in your practice (including yourself)?

[Single answer]

☐ 1 to 5

☐ 6 to 10

☐ 11 to 100

☐ 101 to 500

☐ More than 500

3. During an <u>average week (5 working days)</u>, how many adult patients (≥ 18 years of age) do you evaluate and/or treat face to face in your practice for <u>any</u> medical condition?

[Single answer]

☐ 40 or fewer patients

☐ 41 to 80 patients

☐ 81 to 120 patients

☐ More than 120 patients

4. Over the <u>past month</u>, please provide an estimate of the average number of patients that you have seen in your practice as described below. [Select one.]

[Single answer per row]

		**Estimated number**					
**Patient population**	**None**	**1 to 25**	**26 to 50**	**51 to 75**	**76 to 100**	**> 100**	**I am not sure**
Immunocompromised (e.g., oncology patients, CKD, HIV, etc.)	☐	☐	☐	☐	☐	☐	☐

Chronic liver disease	☐	☐	☐	☐	☐	☐	☐
Current or previous use of illegal drugs (injectable or oral)	☐	☐	☐	☐	☐	☐	☐
Men who have sex with men (MSM)	☐	☐	☐	☐	☐	☐	☐
People with multiple sexual partners	☐	☐	☐	☐	☐	☐	☐
[Show only to GPs] Travelling to a country with endemic levels of hepatitis A infection (e.g., Italy, Turkey, Spain, Russia, South America, Africa, parts of Asia, etc.)	☐	☐	☐	☐	☐	☐	☐
[Show only to GPs] Travelling to a country with endemic levels of hepatitis B infection (e.g., Romania, Latvia, Albania, Southeast Asia, Northern Canada, etc.)	☐	☐	☐	☐	☐	☐	☐
Healthcare workers who may be exposed to hepatitis A in their work duties (e.g., laboratory workers, staff of residential institutions, etc.)	☐	☐	☐	☐	☐	☐	☐
Healthcare workers who may be exposed to hepatitis B in their work duties (e.g., laboratory staff, cleaning staff, nurses, first responders, emergency department staff, etc.)	☐	☐	☐	☐	☐	☐	☐

[Show Q5/Q5a on same screen]

5. Have you ever encountered any adult patients (≥ 18 years of age) who have had or currently have a hepatitis A infection?

[Single answer]

☐ Yes [If yes, display Q5a]

☐ No

☐ I’m not sure

5a. In all of your years of practice, approximately how many adult cases of <u>hepatitis A</u> infection have you encountered?

[Single answer]

☐ 1 to 10

☐ 11 to 20

☐ More than 20

[Show Q6/Q6a on same screen]

6. Have you <u>ever</u> encountered any adult patients (≥ 18 years of age) who have had <u>or</u> currently have a <u>hepatitis B</u> infection?

[Single answer]

☐ Yes [If yes, display Q6a]

☐ No

☐ I’m not sure

6a. In all of your years of practice, approximately how many adult cases of <u>hepatitis B</u> infection have you encountered?

[Single answer]

☐ 1 to 10

☐ 11 to 20

☐ More than 20

[PN: SHOW Q7/Q7a/Q7b/Q7c on same screen]

**7. Do you currently recommend , prescribe, and/or administer the following vaccines in your practice? [This question and the corresponding sequential subquestions cannot be skipped]**	
Hepatitis A	☐ Yes<br>☐ No [if no, display Q7a]
Hepatitis B	☐ Yes<br>☐ No [if no, display Q7b]
Combined hepatitis A/B vaccine	☐ Yes<br>☐ No [if no, display Q7c]

7a. Why do you not recommend, prescribe, and/or administer hepatitis A vaccines? Please select all that apply. [Multiple answers: force at least one response]

- ☐ Hepatitis A infection is very rare in my practice
- ☐ I have only a few patients who are at risk for hepatitis A
- ☐ The reimbursement process is complicated [DE]
- ☐ Some patients are not willing to pay [DE]
- ☐ Many of my patients refuse to be vaccinated for hepatitis A
- ☐ Hepatitis A vaccine is a part of the routine paediatric vaccination calendar
- ☐ Other reasons

7b. Why do you not recommend, prescribe, and/or administer hepatitis B vaccines? Please select all that apply.

[Multiple answers: force at least one response]

- ☐ Hepatitis B infection is very rare in my practice
- ☐ I have only a few patients who are at risk for hepatitis B
- ☐ The reimbursement process is complicated [DE]
- ☐ Some patients are not willing to pay [DE]
- ☐ Many of my patients refuse to be vaccinated for hepatitis B
- ☐ UMV paediatric hepatitis B is in place for my country
- ☐ Other reasons

7c. Why do you not recommend, prescribe and/or administer the combined hepatitis A/B vaccine? Please select all that apply. [Multiple answers: force at least one response]

- ☐ I administer the individual (non-combined) hepatitis A and hepatitis B vaccines
- ☐ Hepatitis A infection is very rare in my practice
- ☐ Hepatitis B infection is very rare in my practice
- ☐ I have only a few patients who are at risk for hepatitis A
- ☐ I have only a few patients who are at risk for hepatitis B

- ☐ There is a lack of adequate reimbursement for hepatitis A/B vaccine administration [UK]
- ☐ The reimbursement process is complicated [DE]
- ☐ Some patients are not willing to pay [DE]
- ☐ Many of my patients refuse to be vaccinated for hepatitis A and/or hepatitis B
- ☐ I am not aware of a hepatitis A/B vaccine recommendation
- ☐ Other reasons

8. Which of the following approaches do you use to inform decision making regarding recommending and/or prescribing <u>hepatitis A</u>, <u>hepatitis B</u>, and <u>combined hepatitis A/B</u> vaccines to patients aged ≥ 18 years? Please select <u>all</u> that apply. [This question cannot be skipped] [Multiple answers: force at least one response]

- ☐ I rely on my own clinical judgement
- ☐ My patients complete a questionnaire in the waiting room or prior to the visit that includes the patient's history of vaccinations
- ☐ I ask and review the history of vaccinations with my patients during the initial visit
- ☐ I identify high-risk individuals (e.g., men who have sex with men, illegal drug users, occupational exposure, travellers to endemic area) based on my evaluation during their visit
- ☐ There have been recent outbreaks of Hep A or Hep B infections in my region
- ☐ I see some patients suffering from Hep A infections
- ☐ I see some patients suffering from Hep B infections
- ☐ I receive training to keep updated on immunisation recommendations and/or guidelines
- ☐ Other (e.g., waiting room posters, television screens, some patients request a vaccine for travel purposes or a job requirement)

Attitudes Toward and Practise Regarding Hepatitis A and B Vaccination

9. How important do you think it is that individuals within the following patient populations should get vaccinated for <u>hepatitis A</u>? [This question cannot be skipped]

Patient population	I do not treat these types of patients	Extremely important	Moderately important	Slightly important	Not important
Chronic liver disease	☐	☐	☐	☐	☐
Immunocompromised (oncology patients, CKD, HIV, etc.)	☐	☐	☐	☐	☐
Current or previous use of illegal drugs (injectable or oral)	☐	☐	☐	☐	☐
Men who have sex with men (MSM)	☐	☐	☐	☐	☐
People with multiple sexual partners	☐	☐	☐	☐	☐
Patients who are food handlers [ES and the UK]	☐	☐	☐	☐	☐
[Show only to GPs] Travelling to a country with endemic levels of hepatitis A infections (e.g., Italy, Turkey, Spain, Russia, South America, Africa, parts of Asia, etc.)	☐	☐	☐	☐	☐
Healthcare workers who may be exposed to hepatitis A in their work duties (e.g., exposure to stools)	☐	☐	☐	☐	☐

10. How important do you think it is that individuals within the following patient populations should get vaccinated for <u>hepatitis B</u>? [This question cannot be skipped]

Patient population	I do not treat these types of patients	Extremely important	Moderately important	Slightly important	Not important
Chronic liver disease	☐	☐	☐	☐	☐

Immunocompromised (oncology patients, CKD, HIV, etc.)	☐	☐	☐	☐	☐
Current or previous use of illegal drugs (injectable or oral)	☐	☐	☐	☐	☐
Men who have sex with men (MSM)	☐	☐	☐	☐	☐
People with multiple sexual partners	☐	☐	☐	☐	☐
[Show only to GPs] Travelling to a country with endemic levels of hepatitis B infection (e.g., Romania, Latvia, Albania, Southeast Asia, Northern Canada, etc.)	☐	☐	☐	☐	☐
Persons at risk for percutaneous, intramuscular, or mucosal exposure to blood (e.g., healthcare and public safety staff, such as first responders or emergency personnel)	☐	☐	☐	☐	☐

11. For each patient population (aged ≥ 18 years) listed, do you RECOMMEND a hepatitis A vaccine? [If a participant selects "I do not treat these types of patients" in Q9, do not display that patient population for this question] [Programming note: this question cannot be skipped]

**Patient population**	**Yes**	**No**
Chronic liver disease	☐	☐
Immunocompromised (oncology patients, CKD, HIV, etc.)	☐	☐
Current or previous use of illegal drugs (injectable or oral)	☐	☐
Men who have sex with men (MSM)	☐	☐
People with multiple sexual partners	☐	☐
Patients who are food handlers [ES and the UK]	☐	☐
[Show only to GPs] Travelling to a country with endemic levels of hepatitis A infections (e.g., Italy, Turkey, Spain, Russia, South America, Africa, parts of Asia, etc.)	☐	☐
Healthcare workers who may be exposed to hepatitis A in their work duties (e.g., exposure to stools)	☐	☐

12. Among those patients to whom you recommend a <u>hepatitis A</u> vaccine, approximately how many patients do you provide a PRESCRIPTION for the vaccine? *<u>Please provide your best estimate</u>*. [Display only the patient population selected in Q11] Single answer per row: this question cannot be skipped]

**Patient population**	**Almost all/All**	**Up to three-quarters**	**Up to one-half**	**Up to one-quarter**	**None/ A few**	**I am not sure**
Chronic liver disease	☐	☐	☐	☐	☐	☐
Immunocompromised (oncology patients, CKD, HIV, etc.)	☐	☐	☐	☐	☐	☐
Current or previous use of illegal drugs (injectable or oral)	☐	☐	☐	☐	☐	☐
Men who have sex with men (MSM)	☐	☐	☐	☐	☐	☐
People with multiple sexual partners	☐	☐	☐	☐	☐	☐
Patients who are food handlers [ES and the UK]	☐	☐	☐	☐	☐	☐
[Show only to GPs] Travelling to a country with endemic levels of hepatitis A infections (e.g., Italy, Turkey, Spain, Russia, South America, Africa, parts of Asia, etc.)	☐	☐	☐	☐	☐	☐
Healthcare workers who may be exposed to hepatitis A in their work duties (e.g., exposure to stools)	☐	☐	☐	☐	☐	☐

13. Among those patients receiving a prescription for a <u>hepatitis A</u> vaccine, approximately how many GET VACCINATED with the vaccine in your practice or at another location? *Please provide your best estimate*. [Display only the patient population selected in Q11] Single answer per row: this question cannot be skipped]

**Patient population**	**Almost all/ All**	**Up to three-quarters**	**Up to one-half**	**Up to one-quarter**	**None/ A few**	**I am not sure**
Chronic liver disease	☐	☐	☐	☐	☐	☐

Immunocompromised (oncology patients, CKD, HIV, etc.)	☐	☐	☐	☐	☐	☐
Current or previous use of illegal drugs (injectable or oral)	☐	☐	☐	☐	☐	☐
Men who have sex with men (MSM)	☐	☐	☐	☐	☐	☐
People with multiple sexual partners	☐	☐	☐	☐	☐	☐
Patients who are food handlers [ES and the UK]	☐	☐	☐	☐	☐	☐
[Show only to GPs] Travelling to a country with endemic levels of hepatitis A infections (e.g., Italy, Turkey, Spain, Russia, South America, Africa, parts of Asia, etc.)	☐	☐	☐	☐	☐	☐
Healthcare workers who may be exposed to hepatitis A in their work duties (e.g., exposure to stools)	☐	☐	☐	☐	☐	☐

14. Why would you not recommend a hepatitis A vaccine for [This question should be displayed to any participant who selects "No" for each of the patient populations in Q11. Display the question for each patient population selected and modify the text to ensure it reads properly {e.g., Why would you not recommend a hepatitis A vaccine for patients with chronic liver disease?; immunocompromised (oncology patients, CKD, HIV, etc.) patients, patients who currently or previously use illegal drugs (injectable or oral), for patients who are men who have sex with men, people with multiple sexual partners, patients travelling to a country with endemic levels of hepatitis A infections (e.g., Italy, Turkey, Spain, Russia, South America, Africa, parts of Asia, etc.), Patients who are food handlers [ES and the UK], healthcare workers who may be exposed to hepatitis A in their work duties (e.g., exposure to stools)}; multiple answers: this question cannot be skipped]?

Please select all that apply. [Force at least one response]

☐ I think the risk of hepatitis A infection is low in this patient population

☐ I am uncertain about what the guidelines say about vaccinating this population

☐ I do not routinely ask my patients if they [Display the description of the patient populations {i.e., have chronic liver disease; are immunocompromised; use illegal drugs; are male and have sex with men (MSM); have multiple sexual partners; GPs ONLY: are travelling to a country with endemic levels of hepatitis A infections; ES and the UK: are food handlers; are healthcare workers}]

☐ I would refer that patient elsewhere (e.g., pharmacy) [UK]

☐ Some of my patients cannot afford to pay for the vaccine [DE and the UK]

☐ The reimbursement from payers (e.g., Gesetzliche Krankenversicherung (GKV), Private Krankenversicherung (PKV) [DE ONLY]_, National Health Service (NHS) [UK ONLY]) for this vaccine is inadequate

☐ The reimbursement process for my patients is complex or not known [DE and the UK]

☐ Patients must pay out-of-pocket for any vaccination because of travel [DE]

☐ I would recommend this vaccine for some of my patients but not all of my patients who [Display the description of the patient populations (i.e., have chronic liver disease; are immunocompromised; use illicit drugs; are male and have sex with men (MSM); GPs ONLY: are travelling to a country with endemic levels of hepatitis A infections; ES and the UK: are food handlers; are healthcare workers)].

☐ Other reasons

15. For each patient population (aged ≥ 18 years) listed, do you RECOMMEND a hepatitis B vaccine [If a participant selects "I do not treat these types of patients" in Q10, do not display that patient population for this question; Single answer: this question cannot be skipped]?

**Patient population**	**Yes**	**No**
Chronic liver disease	☐	☐
Immunocompromised (oncology patients, CKD, HIV, etc.)	☐	☐
Current or previous use of illegal drugs (injectable or oral)	☐	☐
Men who have sex with men (MSM)	☐	☐
People with multiple sexual partners	☐	☐
[Show only to GPs]	☐	☐

Travelling to a country with endemic levels of hepatitis B infection (e.g., Romania, Latvia, Albania, Southeast Asia, Northern Canada, etc.)		
Persons at risk for percutaneous, intramuscular, or mucosal exposure to blood (e.g., healthcare and public safety staff, such as first responders or emergency personnel)	☐	☐

16. Among those patients to whom you recommend a <u>hepatitis B</u> vaccine, approximately how many patients do you provide a PRESCRIPTION for the vaccine? *Please provide your best estimate*. [Display only the patient population selected in Q15;] Single answer per row: this question cannot be skipped]

Patient population	Almost all/All	Up to three-quarters	Up to one-half	Up to one-quarter	None/ A few	I am not sure
Chronic liver disease	☐	☐	☐	☐	☐	☐
Immunocompromised (oncology patients, CKD, HIV, etc.)	☐	☐	☐	☐	☐	☐
Current or previous use of illegal drugs (injectable or oral)	☐	☐	☐	☐	☐	☐
Men who have sex with men (MSM)	☐	☐	☐	☐	☐	☐
People with multiple sexual partners	☐	☐	☐	☐	☐	☐
[Show only to GPs] Travelling to a country with endemic levels of hepatitis B infection (e.g., Romania, Latvia, Albania, Southeast Asia, Northern Canada, etc.)	☐	☐	☐	☐	☐	☐
Persons at risk for percutaneous, intramuscular, or mucosal exposure to blood (e.g., healthcare and public safety staff, such as first responders or emergency personnel)	☐	☐	☐	☐	☐	☐

17. **Among those patients receiving a prescription for a hepatitis B vaccine, approximately how many GET VACCINATED with the vaccine in your practice or at another location?** ***Please provide your best estimate*.** **[Display only the patient population selected in Q15;] [Single answer per row: this question cannot be skipped]**

**Patient population**	**Almost all/All**	**Up to three-quarters**	**Up to one-half**	**Up to one-quarter**	**None/ A few**	**I am not sure**
Chronic liver disease	☐	☐	☐	☐	☐	☐
Immunocompromised (oncology patients, CKD, HIV, etc.)	☐	☐	☐	☐	☐	☐
Current or previous use of illegal drugs (injectable or oral)	☐	☐	☐	☐	☐	☐
Men who have sex with men (MSM)	☐	☐	☐	☐	☐	☐
People with multiple sexual partners	☐	☐	☐	☐	☐	☐
[Show only to GPs] Travelling to a country with endemic levels of hepatitis B infection (e.g., Romania, Latvia, Albania, Southeast Asia, Northern Canada, etc.)	☐	☐	☐	☐	☐	☐
Persons at risk for percutaneous, intramuscular, or mucosal exposure to blood (e.g., healthcare and public safety staff, such as first responders or emergency personnel)	☐	☐	☐	☐	☐	☐

[Force at least one response]

18. **Why would you <u>not</u> recommend a <u>hepatitis B</u> vaccine for [This question should be displayed to any participant who selects "No" for each of the patient populations in Q15. Display the question for each patient population selected and modify the text to ensure it reads properly {e.g., Why would you not recommend a hepatitis B vaccine for patients with chronic liver disease?, ; immunocompromised (oncology patients, CKD, HIV, etc.) patients, patients who currently or previously use illegal drugs (injectable or oral); patients who are men who have sex with men?, people with multiple sexual partners, patients travelling to a country with endemic levels of hepatitis B infection (e.g., Romania, Latvia, Albania, Southeast Asia, Northern Canada, etc.); persons at risk for percutaneous, intramuscular, or mucosal exposure to blood (e.g., healthcare and public safety staff, such as first responders or emergency personnel); Multiple answers: This question cannot be skipped]?**

Please select <u>all</u> that apply.

- ☐ I think the risk of hepatitis B infection is low in this patient population
- ☐ I am uncertain about what the recommendations say about vaccinating this population
- ☐ I do not routinely ask my patients if they [Display the description of the patient populations {e.g., have chronic liver disease; use illegal drugs; are men and have sex with men; have multiple sexual partners; GPS ONLY: are travelling to a country with endemic levels of hepatitis B infections; are at risk for percutaneous, intramuscular, or mucosal exposure to blood}]
- ☐ I would refer that patient elsewhere (e.g., to a specialist physician, pharmacy, public health department, senior centre, workplace) [DE and the UK]
- ☐ Some of my patients cannot afford to pay for the vaccine [DE and the UK]
- ☐ The reimbursement from payers (e.g., Gesetzliche Krankenversicherung (GKV), Private Krankenversicherung (PKV) [DE ONLY], National Health Service (NHS) [UK ONLY]) for this vaccine is inadequate
- ☐ The reimbursement process for my patients is complex or not known [DE and the UK]
- ☐ Patients must pay out-of-pocket for any vaccination due to travel [DE]
- ☐ I would recommend this vaccine for some of my patients but not all of my patients who [Display the description of the patient populations {e.g., have chronic liver disease; use illegal drugs; are men and have sex with men; have multiple sexual partners; GPs ONLY: are travelling

to a country with endemic levels of hepatitis B infections; are at risk for percutaneous, intramuscular, or mucosal exposure to blood}]

- [ ] Other reasons

19. In addition to recommendations, what other factors do you consider before vaccinating patients with a hepatitis A vaccine? Please select all that apply. [This question cannot be skipped] [Force at least one response; multiple answers]

- [ ] Age
- [ ] Willingness/motivation to be vaccinated
- [ ] Medical history (including comorbidities)
- [ ] Vaccination history (documented or patient reported from memory)
- [ ] Other factors

20. In addition to recommendations, what other factors do you consider before vaccinating a patient with a hepatitis B vaccine? Please select all that apply. [This question cannot be skipped] [Force at least one response; multiple answers]

- [ ] Age
- [ ] Willingness/motivation to be vaccinated
- [ ] Medical history (including comorbidities)
- [ ] Vaccination history (documented or patient reported from memory)
- [ ] Other factors

21. In addition to recommendations, what other factors do you consider before vaccinating patients with a combined hepatitis A/B vaccine? Please select all that apply. [This question cannot be skipped] [Force at least one response; multiple answers]

- [ ] Age
- [ ] Willingness/motivation to be vaccinated
- [ ] Medical history (including comorbidities)
- [ ] Vaccination history (documented or patient reported from memory)
- [ ] Reimbursement to the patient (insurance or self-pay) [UK]

- ☐ Patient preference (less administrations with the combined Hep A and B vaccine)
- ☐ Other factors

Knowledge About Hepatitis A and B Vaccination Recommendations

22. Are you familiar with [UK: the Joint Committee on Vaccination and Immunisation and the NHS; DE: Recommendations of the Standing Vaccination Commission (STIKO); ES: Ministry of Health Recommendations]? [This question cannot be skipped]

- ☐ Yes
- ☐ No

23. When making a decision on recommending, prescribing, or administering a <u>hepatitis A</u>, <u>hepatitis B</u>, or <u>combined hepatitis A/B</u> vaccine to adults (≥ 18 years of age), what resource(s) do you use? Please select <u>all</u> that apply. [Multiple answers: this question cannot be skipped]

- ☐ Ministry of Health Recommendations [ES only]
- ☐ The Joint Committee on Vaccination and Immunisation and the NHS [UK only]
- ☐ Recommendations of the Standing Vaccination Commission (STIKO) [DE only]
- ☐ World Health Organization
- ☐ USA Centers for Disease Control and Prevention
- ☐ I do not use any of these resources [Exclusive]
- ☐ Other

**24. When making a decision on recommending or prescribing a hepatitis A, hepatitis B or combined hepatitis A/B vaccine to adults (≥ 18 years of age), to what extent do you follow the [UK: the Joint Committee on Vaccination and Immunisation and the NHS Recommendations; DE: Recommendations of the Standing Vaccination Commission (STIKO); ES: Ministry of Health Recommendations]? [Single answer per row: Programming note: this question cannot be skipped]**	
Hepatitis A vaccine	☐ Always<br>☐ Most of the time<br>☐ About half of the time<br>☐ Some of the time<br>☐ Never
Hepatitis B vaccine	☐ Always<br>☐ Most of the time<br>☐ About half of the time<br>☐ Some of the time<br>☐ Never
Combined hepatitis A/B vaccine	☐ Always<br>☐ Most of the time<br>☐ About half of the time<br>☐ Some of the time<br>☐ Never

25. How likely are you to recommend a hepatitis A vaccine to each of the following patient populations? [If a participant selects "I do not treat these types of patients" in Q9, do not display that patient population for this question; this question cannot be skipped]

**Patient population**	**Very likely**	**Somewhat likely**	**Less likely**	**Unlikely**
Chronic liver disease	☐	☐	☐	☐
Immunocompromised (oncology patients, CKD, HIV, etc.)	☐	☐	☐	☐
Current or previous use of illegal drugs (injectable or oral)	☐	☐	☐	☐
Men who have sex with men (MSM)	☐	☐	☐	☐
People with multiple sexual partners	☐	☐	☐	☐
Patients who are food handlers [ES and the UK]	☐	☐	☐	☐
[Show only to GPs]	☐	☐	☐	☐

Travelling to a country with endemic levels of hepatitis A infections (e.g., Italy, Turkey, Spain, Russia, South America, Africa, parts of Asia, etc.)				
Healthcare workers who may be exposed to hepatitis A in their work duties (e.g., exposure to stools)	☐	☐	☐	☐

26. To what degree are each of the following a barrier or a concern related to <u>RECOMMENDING</u> a <u>hepatitis A</u> vaccine to your patients at risk of infection? [This question cannot be skipped]

	Not a barrier	Minor barrier	Moderate barrier	Major barrier
Not enough time during the visit to assess risk factors	☐	☐	☐	☐
Patient does not disclose risk factors (e.g., use of illegal drugs, sexual practises) to healthcare provider	☐	☐	☐	☐
Your own concerns related to the effectiveness of the vaccine	☐	☐	☐	☐
It is difficult to ensure that patients complete the designated number of doses (2) in the series	☐	☐	☐	☐
Patient's vaccination history cannot be confirmed	☐	☐	☐	☐
Lack of clarity of [UK: the Joint Committee on Vaccination and Immunization and the NHS Recommendations; DE: the Recommendations of the Standing Vaccination Commission (STIKO); ES: the Ministry of Health Recommendations], or other vaccine guidelines	☐	☐	☐	☐

27. To what degree are each of the following a barrier or a concern related to <u>ADMINISTERING</u> a <u>hepatitis A</u> vaccine to your patients at risk of infection? [This question cannot be skipped]

	Not a barrier	Minor barrier	Moderate barrier	Major barrier
Patient's concern for the safety of the vaccine	☐	☐	☐	☐

Patient's lack of motivation/willingness or knowledge of the risk of disease	☐	☐	☐	☐
Patient refuses due to the out-of-pocket cost of vaccine	☐	☐	☐	☐
Patient prefers to get the vaccine elsewhere (work, public health department, senior centre)	☐	☐	☐	☐
Patients may not complete the designated number of doses (2) in the series	☐	☐	☐	☐

28. How likely are you to recommend a hepatitis B vaccine to each of the following patient populations? [If a participant selects "I do not treat these types of patients" in Q10, do not display that patient population for this question; this question cannot be skipped]

**Patient population**		**Very likely**	**Somewhat likely**	**Less likely**	**Unlikely**
Chronic liver disease	☐	☐	☐	☐	☐
Immunocompromised (oncology patients, CKD, HIV, etc.)	☐	☐	☐	☐	☐
Current or previous use of illegal drugs (injectable or oral)	☐	☐	☐	☐	☐
Men who have sex with men (MSM)	☐	☐	☐	☐	☐
People with multiple sexual partners	☐	☐	☐	☐	☐
[Show only to GPs] Travelling to a country with endemic levels of hepatitis B infection (e.g., Romania, Latvia, Albania, Southeast Asia, Northern Canada, etc.)	☐	☐	☐	☐	☐
Persons at risk for percutaneous, intramuscular, or mucosal exposure to blood (e.g., healthcare and public safety staff, such as first responders or emergency personnel)	☐	☐	☐	☐	☐

29. To what degree are each of the following a barrier or a concern related to <u>RECOMMENDING</u> <u>hepatitis B</u> vaccine to your patients at risk of infection? [This question cannot be skipped]

	Not a barrier	Minor barrier	Moderate barrier	Major barrier
Not enough time during the visit to assess risk factors	☐	☐	☐	☐
Patient does not disclose risk factors (e.g., use of illegal drugs, sexual practises) to healthcare provider	☐	☐	☐	☐
Your own concerns related to the effectiveness of the vaccine	☐	☐	☐	☐
It is difficult to ensure that patients complete the designated number of specified doses in the series	☐	☐	☐	☐
Patient's vaccination history cannot be confirmed	☐	☐	☐	☐
Lack of clarity of [UK: the Joint Committee on Vaccination and Immunization and the NHS Recommendations; DE: the Recommendations of the Standing Vaccination Commission (STIKO); ES: the Ministry of Health Recommendations] or other vaccine guidelines	☐	☐	☐	☐

30. To what degree are each of the following a barrier or a concern related to <u>ADMINISTERING</u> a <u>hepatitis B</u> vaccine to your patients at risk of infection? [This question cannot be skipped]

	Not a barrier	Minor barrier	Moderate barrier	Major barrier
Patient's concern for the safety of the vaccine	☐	☐	☐	☐
Patient's lack of motivation/willingness or knowledge of the risk of disease	☐	☐	☐	☐
Patient refuses due to the out-of-pocket cost of vaccine	☐	☐	☐	☐
Patient prefers to get the vaccine elsewhere (work, public health department, senior centre)	☐	☐	☐	☐

31. When you prescribe a hepatitis A or hepatitis B vaccine to a patient, do you have knowledge if the patient actually receives the vaccination?

- ☐ Yes, for **some** patients I have documentation
- ☐ Yes, for **most** patients I have documentation
- ☐ Yes, for **all** patients I have documentation
- ☐ No

HCP's Profile

Now, we would like to know a little more about you.

32. Are you…?

- ☐ Female
- ☐ Male
- ☐ Genderfluid
- ☐ Non-binary
- ☐ A gender identity not listed
- ☐ I prefer not to answer

33. How old are you?

- ☐ 18 to 29 years
- ☐ 30 to 39 years
- ☐ 40 to 49 years
- ☐ 50 to 59 years
- ☐ 60 to 69 years
- ☐ 70 years or older
- ☐ I prefer not to answer

34. Overall, how many years have you been practising medicine as a [Insert response from S3]?

- ☐ 5 years or less
- ☐ 6 to 10 years
- ☐ 11 to 15 years
- ☐ 16 to 20 years
- ☐ 21 to 25 years
- ☐ More than 25 years

35. What is your vaccination status regarding any type of vaccine for yourself as per local recommendations? [This question cannot be skipped] [Single answer]

- ☐ I am fully vaccinated with <u>all</u> recommended vaccines (e.g., influenza, DTP, Covid, etc.)
- ☐ I am partially vaccinated (received greater than 50% of the recommended vaccines)
- ☐ I am not fully vaccinated (received less than 50% of the recommended vaccines)

Thank you for completing the questionnaire!
